# Fly Ash-Based Eco-Efficient Concretes: A Comprehensive Review of the Short-Term Properties

**DOI:** 10.3390/ma14154264

**Published:** 2021-07-30

**Authors:** Mugahed Amran, Roman Fediuk, Gunasekaran Murali, Siva Avudaiappan, Togay Ozbakkaloglu, Nikolai Vatin, Maria Karelina, Sergey Klyuev, Aliakbar Gholampour

**Affiliations:** 1 Department of Civil Engineering, College of Engineering, Prince Sattam Bin Abdulaziz University, Alkharj 16273, Saudi Arabia; 2 Department of Civil Engineering, Faculty of Engineering, Amran University and IT, Quhal, Amran 9677, Yemen; 3 Polytechnic Institute, Far Eastern Federal University, 690922 Vladivostok, Russia; roman44@yandex.ru; 4 School of Civil Engineering, SASTRA Deemed to be University, Thanjavur 61340, India; murali@civil.sastra.ac.in; 5 Departamento de Ingeniería en Obras Civiles, Universidad de Santiago de Chile, Av. Ecuador 3659, Chile; siva.avudaiappan@usach.cl; 6 Ingram School of Engineering, Texas State University, San Marcos, TX 78666, USA; togay.oz@txstate.edu; 7 Moscow Automobile and Road Construction University, 125319 Moscow, Russia; vatin@mail.ru; 8 Department of Machinery Parts and Theory of Mechanisms, Moscow Automobile and Road Construction University, 125319 Moscow, Russia; karelinamu@mail.ru; 9 Department of Theoretical Mechanics and Strength of Materials, Belgorod State Technological University Named after V.G. Shukhov, 308012 Belgorod, Russia; klyuyev@yandex.ru; 10 College of Science and Engineering, Flinders University, Tonsley, SA 5042, Australia; aliakbar.gholampour@flinders.edu.au

**Keywords:** alkali-activated material, fly ash, short-term strengths, pozzolanic, properties, utilizations

## Abstract

Development of sustainable concrete as an alternative to conventional concrete helps in reducing carbon dioxide footprint associated with the use of cement and disposal of waste materials in landfill. One way to achieve that is the use of fly ash (FA) as an alternative to ordinary Portland cement (OPC) because FA is a pozzolanic material and has a high amount of alumina and silica content. Because of its excellent mechanical properties, several studies have been conducted to investigate the use of alkali-activated FA-based concrete as an alternative to conventional concrete. FA, as an industrial by-product, occupies land, thereby causing environmental pollution and health problems. FA-based concrete has numerous advantages, such as it has early strength gaining, it uses low natural resources, and it can be configurated into different structural elements. This study initially presents a review of the classifications, sources, chemical composition, curing regimes and clean production of FA. Then, physical, fresh, and mechanical properties of FA-based concretes are studied. This review helps in better understanding of the behavior of FA-based concrete as a sustainable and eco-friendly material used in construction and building industries.

## 1. Introduction

Concrete as a construction material is extensively used in the construction industry, with global annual consumption of approximately 25 billion cubic meters [1]. Such high usage is due to the concrete’s low cost, excellent durability, readily availability of the constituent materials, and capability to be molded into different shapes [2,3]. Among different constituents of concrete, binder materials are highly important for development of hydration in the microstructure of the concrete [4]. Typically, cement is utilized as a binder in the formation of concrete [2]. It was reported that about 1 ton of CO_2_ is released to the atmosphere for the production of one ton of OPC. It was also reported that annually OPC production releases about 1.5 billion tons of CO_2_ globally, which is corresponding to about 9% of the worldwide total CO_2_ emission globally [5,6,7]. For resolving this global environmental issue of cement production, numerous studies have been performed to find out a sustainable and eco-friendly supplementary cementitious material (SCM) as an alternative to cement in concrete production [8,9,10].

**Figure 1:**
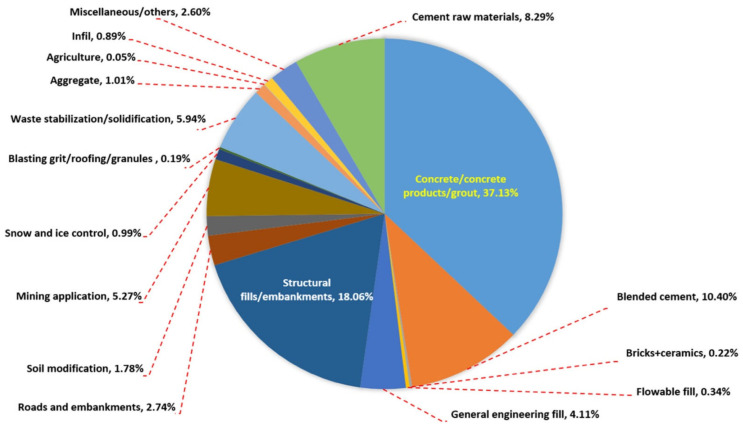
Potential percentage utilization of FA [23]. Reprinted with permission from Elsevier [23].

Among different SCMs used in concrete production, fly ash (FA) an industrial by-product of mineral coal burning made up from fine fuel particles found from flue gases and coal-fired boilers, and it can be employed as an SCM to minimize cement usage in concrete for lowering CO_2_ emissions [8,11,12,13,14,15]. It was reported that more than about 544 million tons of FA are produced annually around the world and 80% of them are discarded in landfill. Moreover, FA has been well known as an eco-friendly material because its utilization reduces carbon footprint of cement production (Figure 1). Using FA at very high content in self-consolidating concrete and high-performance concrete remained limited and further research on the topic is highly needed. Recently, substantial effort has been exerted to develop eco-efficient cement concrete composites [16,17]. FA is called pulverized fuel ash in the UK [18]. The hopper from where the FAs are collected affects their properties, and stockiest interviews showed that the cost of FAs had been already increased by 85–100% in 2012–2016 due to less availability of FA [19]. Despite that, over 6.6 × 10^7^ and 11.1 × 10^7^ tons of FA are generated due to coal-burning for electric power production in the United States and India every year (for example, occupying 26,304 hectares of land in India), respectively. Most FA ends up in landfill or surface impoundments as solid waste, and adequate disposal has been becoming a severe problem. Endeavors to reuse FA wastes were moderately successful, with only about 42% of FA waste being reduced [20,21,22]. Under such a condition, utilizing FA in green application technologies is necessary.

**Figure 2:**
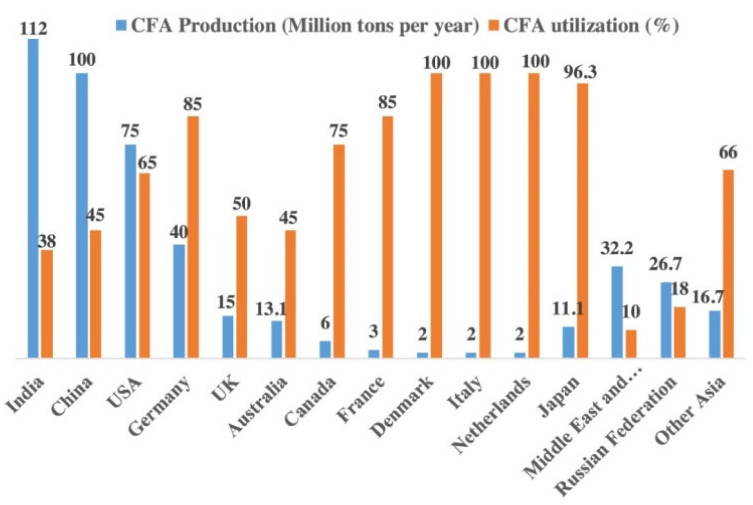
Production and utilization of FA globally (CFA: classified FA) [23]. Reprinted with permission from Elsevier [23].

Recently, substantial efforts have been performed to improve the manufacturing of sustainable cement and the performance of FA-based alkali-activated material (AAM) [16,17]. FA is commonly employed as a pozzolanic material. It is also often employed as OPC’s partial or whole replacement material in concrete production [24]. Endeavors to reuse FA squanders were relatively successful, with about 70% of the total waste being reduced [20,21]. Figure 2 shows the worldwide production and utilization of FA [23]. FA together with other pozzolans is broadly approved by many design codes for utilization as an SCM in concrete, with an FA content of about 55%, according to CEM IV in BS EN 197 [25]. In recent years, researchers have assessed the probability of mixing diverse sorts of wastes with FA [26,27,28,29,30].

**Figure 3:**
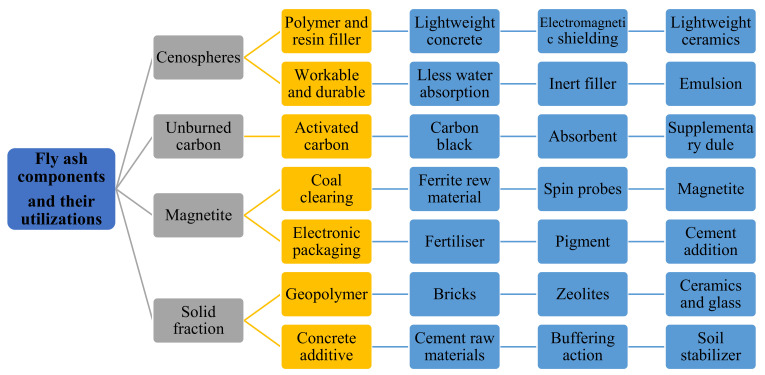
Main constituents of FA and their applications (modified with improvements from Danish [31]).

In many nations, the construction sector demands an increase in the production of SCMs, like FA, because of their role in dropping the CO_2_ emissions caused by cement production. Focus has turned to produce eco-friendly concrete that utilizes by-product materials such as FA (Figure 3) [31,32,33]. An eco-efficient concrete is developed with the use of FA with numerous advantages, which as early strength gaining, low utilization of natural resources, and the ability to configure into different structural elements and to stay flawless for expanded periods without fix works. Therefore, the manufacturing of eco-friendly and economical concrete composite by waste materials received significant interest all around the world. Furthermore, this study presents a review of the classifications, sources, chemical composition, production techniques, curing regimes, and clean production of FA. Subsequently, physical, fresh, and mechanical properties of FA-based concrete are investigated. The aim of this study is to help in better understanding of the behavior of FA-based concrete as a sustainable and eco-friendly material used in construction and building industries.

## 2. Classification of Fly Ash

Based on ASTM C 618, FA is categorized based on its chemical composition into two main classes: Class C and Class F [34,35]. These classes of FA differ mainly in the quantities of four critical constituents in the ash: silica SiO_2_ (35–60%), calcium CaO (1–35%), iron Fe_2_O_3_ (4–20%), and alumina Al_2_O_3_ (10–30%). Moreover, the constituents of FA depend on the particular coal bed makeup. However, FA constituents can also embrace some of the following elements typically found in trace concentrations (up to hundreds of ppm): beryllium, arsenic, boron, cobalt, chromium, cadmium, hexavalent chromium, lead, mercury, manganese, selenium, vanadium, molybdenum, thallium, and strontium, along with low concentrations of dioxins and fragrant polycyclic hydrocarbon composites [36]. According to ASTM standards, FA is classified as Class F if the alumina, silica, and iron contents exceed 70% and Class C if the amounts exceed 50% but lower than 70%.

Class F FA as a pozzolanic material is generally created through bituminous coal and hard and old anthracite, which encompasses less than 7% lime (CaO) [37]. The color of FA is a good indicator of their lime content [36]. For example, dark colors reportedly display a high organic content, whereas light colors mark the existence of high calcium oxide [37]. The alumina and glassy silica of Class F FA, which contains pozzolanic characteristics, demand a cementing agent, such as OPC, hydrated lime, or quicklime, to react and give cementitious compounds. Furthermore, Class F FA can produce an AAM polymer through chemical activators, such as sodium silicate [38,39,40,41]. On the contrary, Class C FA comprises burning coal lignite or younger sub-bituminous that have specific properties of self-cementing [42]. Unlike Class F FA, Class C FA does not require an activator and the sulfate (SO_4_) and alkali contents are more significant than those in Class F FA [43]. The source of SO_3_ and CaO in FA is calcium, and the carbon content is generally not calculated directly but anticipated to be nearly equivalent to loss on ignition (LOI) at 1000 °C [44].

The LOI values of FA must be at lowest possible level and are commonly restricted between 5% and 6% by standards because carbon’s cellular particles raise the demand for air-entraining admixtures, water-reducers, and water in concrete, resulting in a negative effect on the concrete’s mechanical properties, durability, and workability. Table 1 shows the most frequently used specifications for FA and Table 2 tabulated the common properties of FA.

## 3. Source of FA Material

**Figure 4:**
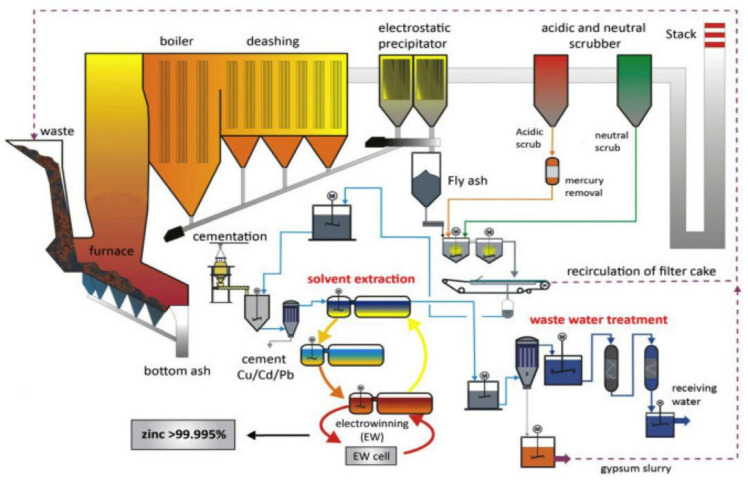
Procedure diagram of incineration fly ash [56]. Reprinted with permission from MDPI [56].

FAs are by-products of burning coal generating electricity in thermal power plants and produces by flue gases using electrostatic precipitators (ESPs) [53]. In practice, geopolymer concrete has two primary constituent materials: (a) dry materials; and (b) alkaline liquids [54]. Geopolymer concretes dry materials are the basis of aluminosilicate, and therefore, have to be rich in aluminum and silicon (Si) [55]. Such dry materials are in the form of natural minerals (e.g., clay, micas, kaolinite, alousite, and spinel). Moreover, the constituents of FA depend on a particular coal bed makeup; yet, they might contain some of the following substances found in trace concentrations (up to hundreds of ppm): crystalline phases of silicon and heterogeneous glassy iron, calcium, aluminum, and magnesium [54]. Also, chemical analysis has indicated that FA compounds serve as the oxides of these elements (Table 3), and they occur as aluminosilicate glass interposed with a small segment of crystalline elements, such as mullite, quartz, magnetite, hematite, and aluminosilicate glass or elemental siliceous [36]. Figure 4 shows the Treatment techniques for incineration fly ash, to determine the effectiveness of obtaining environmentally stable content, and finally, to find potential applications for incineration fly ash based on determining the processing suitability, efficiency, and environmental impact of incineration fly ash for its applications [56].

## 4. Clean Production

**Figure 5:**
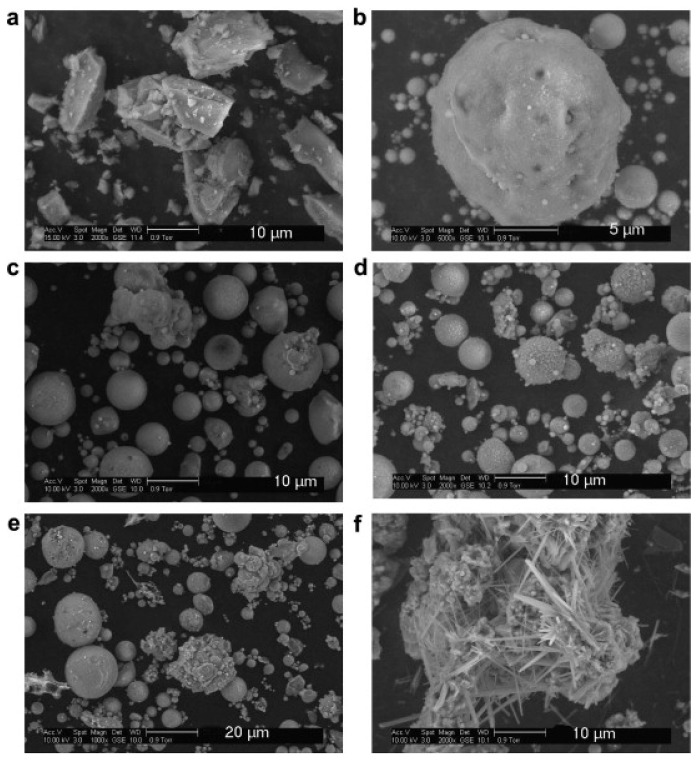
Scanning electron micrographs (SEMs) of cement and fly ash. (**a**) Cement; (**b**) Class C; (**c**) Class F; (**d**) SW1; (**e**) SW2 and (**f**) Wood [66]. Reprinted with permission from Elsevier [66].

**Figure 6:**
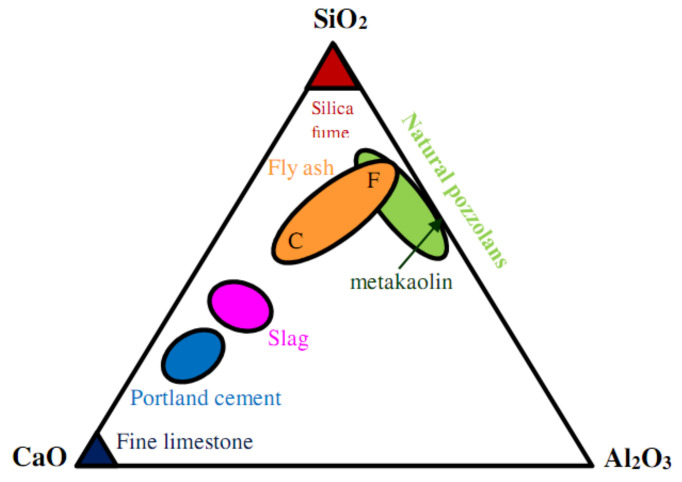
Ternary diagram of supplementary cementitious materials (SCMs) [67]. Reprinted with permission from Elsevier [67].

Generally, the constituents of FA classically include Fe_2_O_3_, SiO_2_, CaO, and Al_2_O_3_; and such compounds exist in the form of crystalline oxides and amorphous in several minerals [64]. FA particles are typically fine like cement and contain glassy rounded particles and residues of magnetite, quartz, hematite, mullite, char, and other crystalline phases formulated while cooling [61]. FA comprises heterogeneous combinations of crystalline and amorphous (glassy) phases [16,24,65]. It is reported that wood fly ash particles have irregular shapes while other ash particles show approximately spherical shapes. as shown in Figure 5 [66]. Moreover, the glassy phases are typically 60–90% of the total mass of FA, with the residual segment of FA composed of diverse crystalline phases [64]. FAs characteristically comprise less calcium and more silicon than OPC and slags, thereby showing that SCMs with high calcium content can have their cementing characteristics. Figure 6 demonstrates the Al_2_O_3_, SiO_2_, and CaO contents in OPC and SCMs [67]. The FA properties vary based on the hopper from where it was accumulated [68]. However, the farthest ESP hopper may have the greatest density, finest particle size, highest glass content, and lowest carbon content [69].

**Figure 7:**
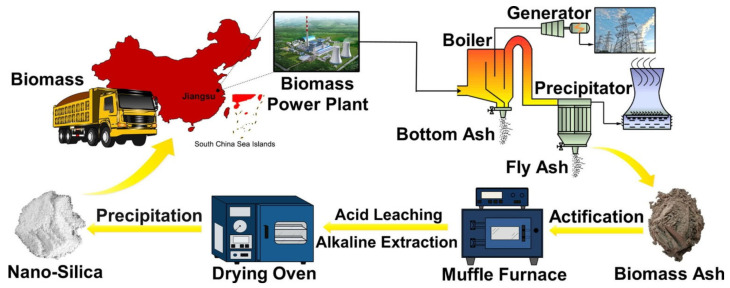
Diagram of clean production of FA (coal → FA) [70]. Reprinted with permission from Elsevier [70].

FA is manufactured from coal combustion in electric utilities or industrial boilers (Figure 7) [70]. In that sense, coal-fired boilers have four main types: fluidized-bed combustion (FBC), pulverized coal (PC), cyclone, and stoker-fired boilers. PC boiler is the most common and is regularly used for outsized electric-generating units, whereas the other boilers are more prevalent in cogeneration or industrial facilities [55]. FA is caught at a time when the exhaust gas stream passes filter fabric collectors (generally in baghouses) or the flue gases through ESPs using a pollution control system (PCS) obtained in a combustion chamber [71]. Depending on the efficacy of the PCS, a minimal amount of FA can be passed to the atmosphere. A dry bottom furnace is the most used coal-burning furnace [72]. About 80% of the total ash leaves, as FA, is available in the exhaust fume when crushed coal is burnt at a dry-bottom boiler [73]. Alternatively, pulverized coal burnt in a wet-bottom furnace will result in 50% of the ash reserved in the furnace while the remainder entrained in the flue gas [19].

On the other hand, in a cyclone furnace, up to 70–80% of the total ash that is retained as boiler slag from the crushed coal is utilized as fuel [74], while merely 20–30% of the total ash leaves the furnace in the flue gas as dry-ash [73]. Table 4 shows the global production and consumption of FA [75,76]. The quality of the produced FA relies on its source and characteristic of the coal being scorched.

**Figure 8:**
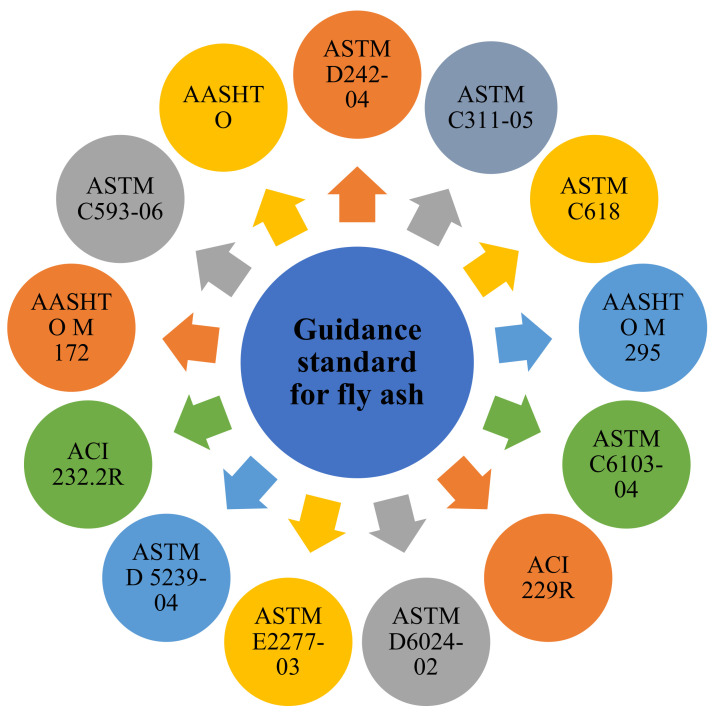
Guidance standards used for FA quality assurance.

Moreover, FA comprises considerable amounts of Al_2_O_3_, SiO_2_ (crystalline and amorphous), and CaO; and these core minerals are constituents in coal-bearing rock strata under different guidance standards used for FA quality assurance (Figure 8). Reportedly, the physical and chemical properties of FA differ based on the source of coal, combustion methods, and the particle shapes [4,17].

## 5. Chemical Composition

FA’s chemical composition relies on the coal source and the boilers’ functioning parameters (Table 5) [72,77,78]. However, as a result of mineral variable sources and processes, mineral admixtures differ considerably in chemical compositions and when added to cement [79]. The chemical properties of FA are extensively influenced by the burnt coal’s chemical content, such as anthracite, bituminous, and lignite [80].

FA materials harden when they are still suspended in exhaust gases and are produced by filter bags or electrostatic precipitators [81]. Furthermore, the particles that harden rapidly while suspended in exhaust gases commonly range from 0.5 µm to 300 µm and are spherical in shape [82,83]. The essential significance of fast cooling is to crystallize certain minerals, while quenched glass and amorphous remain. However, certain refractory stages in pulverized coal remain crystalline and do not dissolve totally [83]. Consequently, FA becomes a heterogeneous material, and Fe_2_O_3,_ Al_2_O_3_, SiO_2_, and sometimes CaO are the principal chemical constituents of different FAs. The mineralogy of FA is highly varied [84]. The critical stages faced are a glass phase composed of mullite, quartz, iron oxides, hematite, maghemite, and magnetite [84,85].

**Table 5:** Chemical composition of FA reported in different studies.

Year	Chemical Composition									Ref.
	Al_2_O_3_	SiO_2_	Fe_2_O_3_	CaO	TiO_2_	K_2_O	SO_3_	Na_2_O	MgO	
2003	31.5	53.7	5.5	2.0	0.7	2.4	0.6	0.8	2.6	[86]
	28.6	61.9	4.3	0.8	1.1	1.3	-	2	-	[87]
2009	24.24	62.79	3.86	1.78	-	-	-	-	1.28	[88]
	20.46	65.64	4.64	2.50	0.36	2.65	0.19	0.60	2.21	[89]
2010	25.95	63.66	2.84	1.19	0.74	2.90	0.25	0.48	0.86	[90]
	20.85	64.64	4.05	2.24	0.31	3.19	0.24	0.93	1.85	
2011	24.67	64.75	3.20	1.01	-	3.09	0.16	0.88	1.64	[91]
2015	16.7	73.1	1.95	1.05	0.35	3.94	-	2.42	-	[92]
2016	33.55	61.24	1.12	0.97	-	0.60	0.30	0.50	0.96	[93]
	26.7	64.4	4.0	3.9	-	-	-	-	1.5	
	28.95	47.19	12.59	5.17	1.06	2.24	0.00	2.27	0.15	
	21.49	64.61	2.75	4.85	0.91	1.80	0.00	3.34	0.10	
	25.13	49.49	1.99	14.69	0.00	2.23	-	3.12	3.35	[94]
	25.34	65.16	3.43	0.91	-	2.38	0.12	0.41	1.35	[93]
	32.4	60.1	3.6	3.1	-	-	-	-	0.8	
	18.98	60.88	9.97	3.08	0.35	2.73	0.33	0.72	2.11	
	28.9	56.4	11.7	1.6	-	-	-	-	1.5	
	25.81	60.45	5.20	0.00	1.56	3.62	-	1.09	2.28	[94]
2017	27.51	51.36	13.05	2.59	1.08	3.16	-	0.53	0.23	[95]
2018	29.70	62.21	3.53	0.90	1.20	1.70		-	-	[96]
2019	55.0	80.0	44.7	52.0	3.7	11.0	-	3.9	15.0	[75]
2020	25.8	55.7	6.9	8.7	-	-	0.6	-	-	[31]

Furthermore, few other stages are frequently documented: free lime, anhydrite, cristobalite, calcite, periclase, halite, sylvite, portlandite, rutile, and anatase [72]. The Ca bearing minerals are gehlenite, anorthite, and akermanite, and several CaAl_2_O_4_ and Ca_2_SiO_2_, such as those present in OPC, are well-known in several Ca-rich types of FAs [68]. The mercury content can be up to 1 ppm and is commonly involved in the range of 0.01–1 ppm for bituminous coal [85,97]. Other trace component concentrations differ by the type of coal burnt to form it [98,99]. Apparently, for bituminous coal, the trace component concentrations are analogous to the trace component concentrations in clean soils, given boron’s remarkable exclusion [98,100].

### Mineralogical Composition

**Figure 9:**
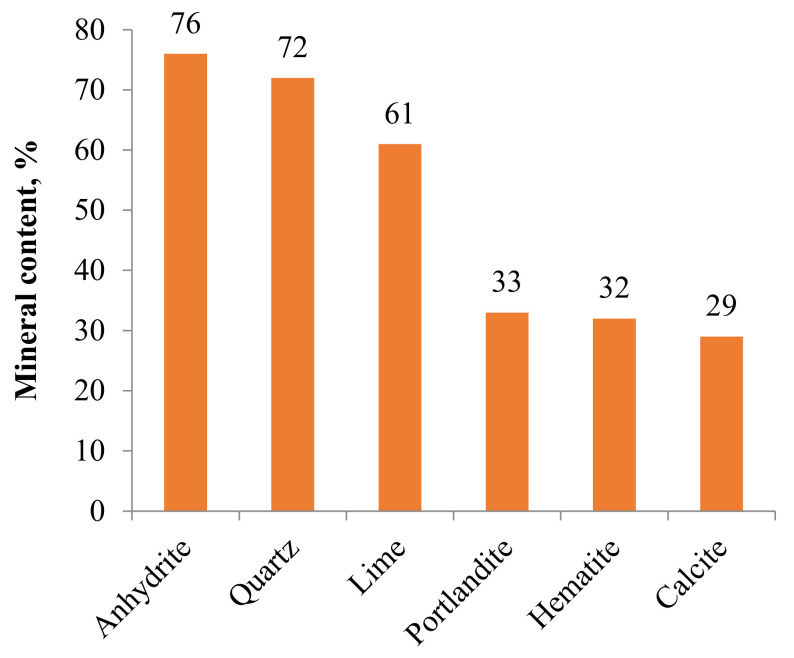
Crystalline phases for different FAs [103]. Reprinted with permission from MDPI [103].

FA’s mineralogical composition is generally affected by its type and source [101]. FAs usually contain a small crystalline material and glass/non-crystalline particles (≤90%) [102]. Certain unburned coals are often composed of ash particles relying on the scheme and burning procedure. Besides, given a considerable volume of glassy material, each FA may include mullite, quartz, magnetite, and hematite (Figure 9) [103,104]. Quartz is naturally a non-reactive material during FA hydration and its content varies from 4% to 23% in FAs [105]. Quartz with crystallite typically has a size that exceeds 125 μm, and its clean structure is mainly from the coal named primary quartz. The next quartz is generally shaped from cooled ash after combustion; thereby, highlighting large matrix parameters and crystallite sizes not exceeding 125 μm. Thus, the crystalline mineral content is within the range of 11–48% [57]. Mullite is mainly created through coal combustion by disintegrated clays, and its crystal composition is shaped in the cooling stage [104,106]. Mullite has low reactivity and thus does not contribute to the hydration process. The hematite and magnetite contents in all FAs are typically limited to less than 5% [104]. In sub-bituminous FAs, the crystalline stages may involve alkali and calcium sulfates [107]. The reactivity of FAs is associated with the noncrystalline stage and glass. High calcium FA is inclined to have minimal mullite content limited to 0.86–1.14% considering low initial alumina content or a high possibility of alumina to form feldspar and tricalcium aluminate [108]. Furthermore, a low-calcium FA comprises nearly 3–24% mullite [57,105,109]. The glass’s chemical composition may prompt the high reactivity of high-calcium FAs. According to previous research findings, more than 188 mineral groups and 316 individual minerals are recognized in FAs [101,110], and the glass composition in high- and low-calcium FAs differ from each other. The mineral composition is commonly more multifaceted in high-calcium FAs, which has been identified to have large volumes of crystalline C_3_A (4–8%), CaO (1–2.5%), and C_4_A_3_S (1–2.5%) than in low-calcium FAs [111]. Furthermore, the large size segment of FA, within 45 μm to 75 μm, is commonly supplemented with first quartz [104]. Besides, the anhydrite content (limited to 10% in high-calcium FAs) is reasonably high because the coal that produces high-calcium FAs has high sulfur content [59].

## 6. Typical Curing Regimes of Fly Ash-Based Concrete

The typical curing regimes (water and steam curing) are given in detail in this section to help understanding the impact of curing on the hardened state of FA-based concrete.

### 6.1. Water Curing

Curing protects concrete from evaporation, temperature extremes, and the negative influence of cement hydration [77]. Fresh concrete must have adequate water content for hydration process to gain potential strength, improve durability performance, and maintain chemical reactions at a rapid and continuous rate [78]. After concrete casting, each test specimen must be stored in the casting room at approximately 30 °C to be later demolded after 24 h for water curing [112]. In water curing, sufficient time is given and the concrete gains its strength rapidly between 3 and 7 days; thereby, achieving the desired strength prescribed in design codes [82]. Curing time and temperature are the most influential factors in FA-based AAM’s compressive strength. However, Adam [113] stated that AAM could solidify quickly at room temperature and exhibited compressive strength of at least 20 MPa after 4.5 h at 20 °C and approximately 70–100 MPa after 28 days. A group of researchers performed tests on AAM mortars and found that the maximum strength of FA-based AAM is achieved in the first two days of curing [114]. Reportedly, elevated temperature curing enhances the strength by removing water from the FA-based AAM; thereby, initiating the failure of capillary pores in a dense structure [115]. FA-based AAM could be remedied at room temperature, but its strength grows gradually and continuously, and thereby, requires extended curing time [116]. Nasvi et al. [117] discovered that the crack initiation thresholds and crack closure of FA-based AAM cured at high temperatures (60 °C–80 °C) were higher (30–60%) than those remedied at air temperature (23 °C and 40 °C). Nonetheless, sustained curing at elevated temperatures disrupted AAMs’ rough composition, causing dehydration and extreme shrinkage and lowering the desired strength [118].

### 6.2. Steam Curing

Steam curing is a standard heating method by transmitting heat to the FA-based AAM paste through steam. The heating varies and requires an extended time to achieve the desired temperature. Microwave heating depends on the inner energy debauchery related to molecular dipoles excitation in electromagnetic fields and conveys quick and constant heat [119]. In steam curing, concrete is permitted to dry in the air with strength of about 50% of that of moist-cured concrete after water curing for 28 days from casting [77,78,112]. The test can be performed in line with BS 1881: Part 110 [120]. The AAM that is cured in the absence of high heat can be utilized in other areas beyond precast members [121]. Manesh et al. [122] prepared FA-based AAM pastes with sodium silicate (Na_2_SiO_3_) solution and 10 mol/L sodium hydroxide (NaOH) remedied for 5 min below 90 W microwave radiation by further heating at 65 °C (6 h), and compared the compressive strength with FA-based AAM cured at 65 °C (24 h). Radiation of microwave creates dense microstructure, accelerates the FA dissolution in an alkali solution, and reduces the curing time [123]. Moreover, AAM can attain higher compressive strengths through oven curing compared to that of ambient curing [124].

## 7. Physical Properties

FA’s physical properties are significantly affected by their particle size. Table 6 shows the physical properties of FA obtained from different studies.

### 7.1. Density

It was reported that, typically, FAs have an average diameter of less than 10 μm, which makes them very fine particles [52]. In addition, FAs are considered to have a high surface area and a low-to-medium bulk density [125]. The density of FA is a crucial parameter because it affects the permeability, compressibility and strength of FA [36].

**Table 6:** Physical properties of FA.

Properties	The Range (Average)	Ref. [126]	Ref. [127]	Ref. [128]	Ref. [129]	Ref. [130]	Ref. [131]	Ref. [23]
Density (g/cm^3^)	0.9–2.6	˂1.65	2.17	1.2–2.23	1.9–2.55	2.30	1–1.7	2.03
Bulk Density (g/cm^3^)	0.5–1.7	~1.23	1.26	0.99	1.6–1.8	0.57 –1.7	0.54–0.86	0.60–1.8
Particle Shape (µm)	Spherical/Irregular	Spherical	Spherical/Irregular	Spherical				
Average particle size (μm)	0.5–300	˃150	1–150	6.92	10–100	~170	0.5–300	10–100
Color	Grey/Dark Brown/tan	Grey	Grey/Dark	Whitish grey	Tan–light	Tan–gray	Brown/grey	White
Specific gravity (g/cm^3^)	1.90–3.20	2.23	2.18	2.29	2.25–3.15	1.9–2.55	2.1–3.0	1.8–2.1
Pozzolanic activity index at 28 days (%)	75–100	79.9	˂75	-	80	80–95	75	75
Fineness, passing 45 µm (%)	12–55	83.2	32.5–52.5	˃53	34	12.5	34	40
Soundness, Le-Chatelier (mm)	10	-	10	-	10	10	10	10

Furthermore, compaction could be used to increase the density of FA by reducing the volume of air [77]. The compacted unit weight relies on the material properties (i.e., particle shape, moisture content at compaction, gradation, and plasticity) and the method and amount of energy application. The maximum density of FAs differs from 0.92 g/cm^3^ to 1.42 g/cm^3^, and their moisture content varies from 18% to 45% under the standard Proctor compaction effort [132]. Low densities are attributed to the low specific gravity (SG) of FA.

Curing can significantly influence FA-based AAMs’ strength by varying the density and porosity of the AAM [133]. The density and pore structure of FA-based AAMs facilitate moisture discharge and help to avoid failure during heating process. For example, the AAMs activated by sodium hydroxide display a rapid strength weakening and a high shrinkage at a temperature of 800 °C, whereas those activated by KOH displays a substantial upsurge in strength while heating and strength weakening start at around 1000 °C [134]. The inclusion of a foaming agent at low concentration causes porous structures with less density in AAM. Zhu et al. [135] made ceramic foams by adding 40% FA, and the sintered foams exhibited a bulk density of 0.46 g/cm^3^, compressive strength of 5 MPa and thermal conductivity of 0.36 W/mK. The increase in density and long-term pozzolanic action of FA has been attributed to free lime, resulting in a few bleed channels and decreased permeability [133]. Furthermore, spherical particles, instead of crushed ones, displayed a higher packing density at the wet state. It was found that FA has a very minimum variation to density due to moisture content comparing to natural soils [136]. FAs can be more insensitive to moisture content variation than other materials considering their high air void content. Voids limit the accumulation of pressure in the pores during compaction with the water content [5,137]. Small and spherical FA particles fill voids or airspaces and increase density [138,139]. These observations reveal that the moisture content in FA can be conveniently controlled in the field if FA is utilized as an embankment fill. The geometric specific surface area can be calculated by Equation (1) assuming that particles are impeccably circle molded [128].

(1) $$\mathrm{Geometric}\;\mathrm{specific}\;\mathrm{surface}\;\mathrm{area}=\frac{\frac{0.6}{D}}{\mathrm{Particle}\;\mathrm{density}}$$

where D is the particle size. Normalized dry unit weight (γ_dn_) can be expressed by Equation (2).

(2) $$\gamma_{\mathrm{dn}}=\gamma_{d}\;\frac{G_{\mathrm{std}}}{G_{m}}$$

where γ_d_ is the dry unit weight of a given material (kN/m^3^); G_m_ is the SG of a given material, and G_std_ is the standard estimation of SG regarding which of the plots are standardized.

### 7.2. Specific Gravity (SG) and Grain Size

The SG is determined in accordance with ASTM D 854. SG of a material depends on several factors, including chemical composition, particle shape, gradation, the proportion of cenospheres, iron-rich magnetite, and unburned carbon particles [97]. In general, the SG of FA typically varies from 2.1 to 3.0, whereas its specific surface area could well change from 170 to 1000 m^2^/kg [132], indicating that a reduction in FA density is mainly due to the decrease in its SG. The SG ranges from smaller values of 1.90 for sub-bituminous ashes to larger values of 2.96 for iron-rich bituminous ashes [140]. Specific sub-bituminous ashes have a reasonably low SG of approximately 2.0, indicating that hollow particles (i.e., plerospheres or cenospheres) are found in substantial amounts in the ashes [141,142,143,144]. Even in the same FA, the SG of coarse particles is minimal given the high carbon content; and considering the low SG, FA has a low unit weight [145]. Furthermore, replacing cement with FA on an equal weight basis increases the paste volume because the SG of FA is smaller than that of cement [146]. Equations (3)–(5) may be used to determine the bulk, submerged, and apparent SGs, respectively. In these equations. A is oven-dried sample weight, B is the submerged specific gravity (SSG) sample weight in air and C is the saturated sample weight.

(3) $$\mathrm{SG}=\frac{A}{\left(B-C\right)}$$

(4) $$\mathrm{SSG}\;\mathrm{Bulk}=\frac{B}{\left(B-C\right)}$$

(5) $$\mathrm{Apparent}\;\mathrm{SG}=\frac{A}{\left(A-C\right)}$$

### 7.3. Strength Activity Index

**Figure 10:**
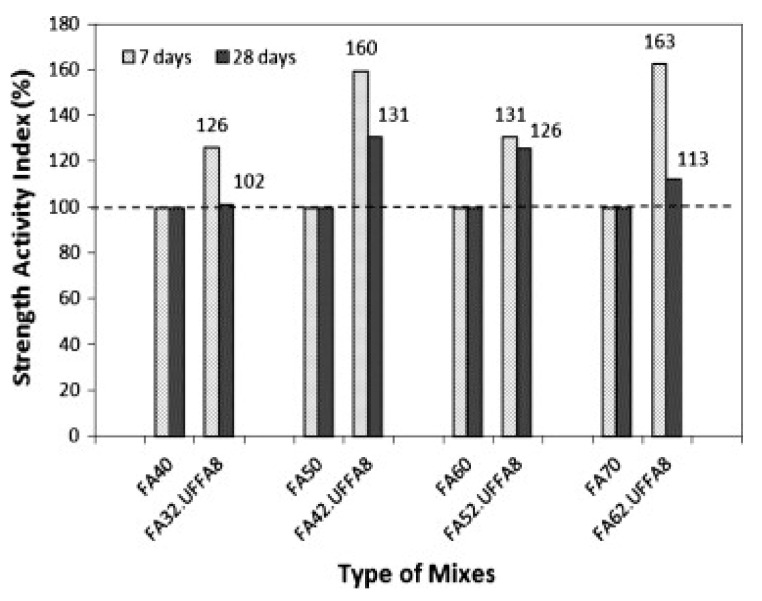
Strength activity index versus type of mixes with ultra-fine FA [151]. Reprinted with permission from Elsevier [151].

The pozzolanic reactivity of a material is quantified by an ancillary method to determine the strength activity indicator [77,78]. The strength activity index (SAI) test is applied to ascertain if the use of FA or natural pozzolan results in an adequate strength development level in concrete [5]. In principle, two test specimens’ sets are used to determine SAI: one is with 100% OPC as a reference and the other with a standardized part of the OPC substituted by the corresponding pozzolan material, as shown in Table 7. The two specimen sets are verified for compressive strength after curing. Therefore, SAI of the mix with pozzolanic material is determined by Equation (6).

(6) $$\mathrm{Strength}\;\mathrm{activity}\;\mathrm{index}\;(\mathrm{SAI})=\frac{\sigma_{\mathrm{Pozzolanic}\;\mathrm{mix}\;}}{\sigma_{\mathrm{Reference}}}$$

where σ is strength. SAI is higher for mixes with fine FA than those with coarse FA [147]. Fine FAs improve mortar workability through improved packing, and reduced water demand caused by the ash particle spherical shape [77,78,148]. It is reported that when FA is used by up to 35%, SAI is only 52.6% [149,150]. It is also found that the most significant improvement is observed in mortar containing 50% fly ash whose 28 days compressive strength is improved by about 31% due to addition of 8% UFFA (Figure 10) [151]. Based on ASTM C 618, the minimum SAI of mix with FA is 75% compared to that of the reference mix at 28 days when 20% of OPC is replaced with FA. This hints that the SAI of insoluble materials depends more on their particle size and less on the curing ages [152]. FA-based concrete with a insoluble material with smaller particle size has a greater SAI than that with larger particle size. SAI remains constant even when FA with large particle size is used because FA with large particle size has low pozzolanic reactivity [152,153]. The acidic oxide content is not a contributing factor for determining SAI of FAs within the acidic oxide content range of 65–68%. SAI differs broadly between 64% and 100%. No significant trend was reported for Class F FA. Furthermore, the FA-based mortar used for measuring SAI in ASTM C 618 is centered on the postulation that FA is 100% as efficient as cement, thereby implying a one-to-one replacement. Equation (7) is applied for calculating SAI in ASTM C 618.

(7) $$C_{\mathrm{equ}}=\left(1-P\%\right)\times \left(C+F\right)+F_{eff}K\times \left(P\%\left(C+f\right)\right)$$

where C_eq_ = cement equivalent, which equals to 500 g; F = mass of FA in g; C = mass of cement in g; *F_eff_* = FA efficiency; and *P*% = percentage of FA. F = 0.25 × C.

### 7.4. Color

**Figure 11:**
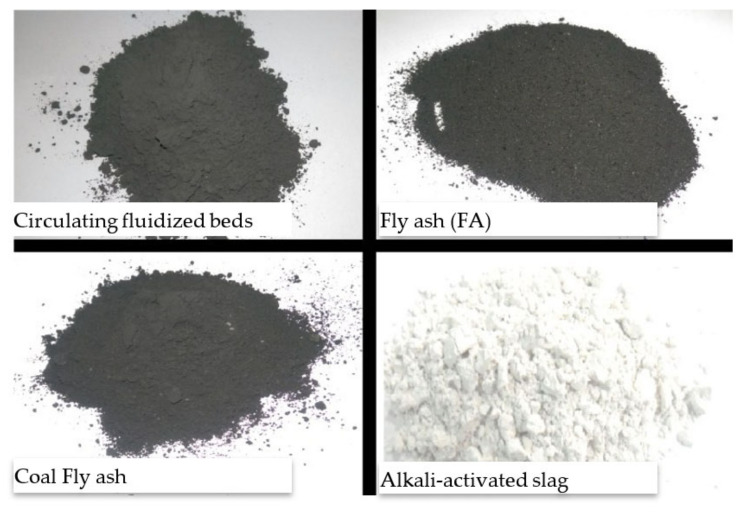
FA with gray to black color compared to the other identical SCMs [8]. Reprinted with permission from Elsevier [8].

Color is a physical property to qualitatively predict the lime content of FA [36]. The color of FA and its influence on the final concrete color may also be necessary [16]. Color is affected by the absorption of water by the formwork material [83]. During the first weeks after concrete casting, a variation in color can be seen while the cement’s hydration continues [83]. In general, fresh FA-based AAM typically displays dark color and a shiny appearance. FA is gray, mainly alkaline, and abrasive, with the pH ranging from 9 to 9.9 [16,154,155]. The color of FA can differ from tan to gray and to black, as shown in Figure 11, based on the chemical and mineral constituents and the quantity of unburned carbon in the ash [36,156,157]. A light color indicates low carbon content, and dark colors suggest a high organic content [37]. Lignite or sub-bituminous FAs are typically light tan to buff in color, thereby signifying reasonably low quantities of carbon and certain calcium or lime availability. Bituminous FAs frequently have a shade of gray, with the light shades of gray commonly signifying a high ash quality [37]. Besides, iron content is usually signified by a brownish color, while unburned content is typically characterized by a dark gray to black color. FA coloring is regular for each coal source and plant. Unburnt carbon and iron contents affect the light color, which varies from brown to opaque, water-white to yellow, or orange to deep red [158].

Furthermore, based on EN 450-1 and ASTM C 618 standards, a rise in LOI lowers the FA quality. For example, larger carbon content could contribute to mixture segregation and concrete discoloration. Moreover, the color of concrete containing FA can change with an increase in temperature [159].

### 7.5. Particle Shape and Size

FA comprises fine and powdery particles that are mainly round in shape [36]. Table 8 shows the typical grain size and grading quality of FA. FA is either hollow or solid, and generally glassy with size of ranging typically 10 to 100 µm depending on the source of material (Table 8) [133]. Specific ashes could be coarser or finer than OPC particles. Furthermore, FA particles are commonly spherical in shape, including cenospheres with 0.2 to 1.1% of the total FA weight. The particle morphologies of FA mainly affect the fluidity of the concrete and are mostly governed by the temperature of combustion, cooling rate, and particle composition [39]. The particle size differs depending on the combustion technique and coal source and is usually limited between 1 µm to 200 µm [39]. Most of FA’s reactive particles have diameters of less than 10 µm and angular particles make up the carbonaceous material in FA [140].

Most bituminous coal FAs have a particle size distribution comparable to that of silt (less than 0.075 mm or No. 200 sieves) [36]. Furthermore, sub-bituminous coal FA is usually slightly coarser than bituminous coal FA, even though the former is in silt size (0.074–0.005 mm). Such particles seem to be solid, while the larger residual particles seem to be parts of hollow, thin spheres comprising much smaller size particles [133]. For example, the N-carboxymethyl chitosan biopolymer coating of FA particles and N-carboxymethyl chitosan are well-combined into the AAM structure [178]. The addition of nano-silica (up to 3%) into FA-based paste results in an increase in the flowability since the spherical shape of nano-silica has a ball-bearing influence on FA particles [179]. The LOI of FAs must be low and usually restricted to 5–6% by standards. The cellular particles of carbon increase the requirement for water in concrete, increasing the amount of water-reducer, which influences the properties of concrete [140].

### 7.6. Fineness

The fineness of FA is important as it affects concrete workability and the rate of its pozzolanic activity. Standards require FA with at least 66% passing the 0.044 mm (No. 325) sieve [77]. Dry- and wet-sieving (ASTM C 311 and ASTM C 430) are frequently used for measuring the fineness of FAs. Hydration rate is also dependent on particles’ fineness [97]. A high fineness is necessary if a quick strength is required [180]. Previous research illustrated that the fineness of FA had a significant effect on the characteristics of hardened or fresh mortar and concrete [181]. The fineness of FA can improve sulfate resistance, lower the expansion, and influence the demand for water and compressive strength of mortar [28].

SAI of finer FAs is larger than that of coarser FAs. Finer FAs improve mortar’s workability because the ash particles’ spherical shape provides enhanced packing, lubricates the paste, and reduces water demand [182]. This is similarly recognized as the ball bearing effect [77]. Although numerous studies examined the effect of ambient condition on the hydration of FA-based pastes, percentage of FA content and water-to-cement (w/c) ratio, only a handful of them evaluated the impact of the chemical composition of FA on concrete. FA’s composition reportedly influences the pore solution’s composition and the hydration kinetics of FA-cement pastes [182]. Furthermore, the fineness of FAs largely relies on the grinding of the coal and the operational settings of coal crushers. A fine gradation usually ends up in reactive ash and comprises minimal carbon. The fineness of FA has been widely reported that has a vital effect on th strength development. The permeability and porosity of pastes are affected by the fineness, shape, and content of FA [183]. The porosity increases with an increase in the replacement level of FA and reduces with a rise in the fineness of FA [28]. Despite a rise in the total porosity of concrete upon incorporating FA, the penetrability decreases given the refinement of pores [184]. It was also reported that the chemical composition of FA has a slight effect, and the fineness of FA has the main impact on the early hydration rate of concrete [185].

### 7.7. Pozzolanic Activity

**Figure 12:**
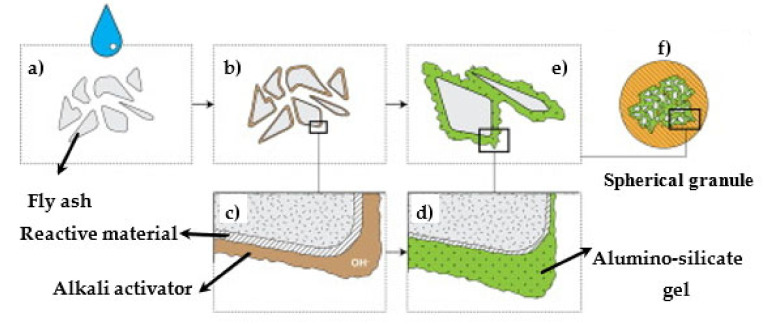
Descriptive prototypical of the granulation-alkali activation technique; (**a**) the precursor particles, (**b**) granulation process, (**c**) Materials reactions, (**d**) aluminosilicate gel, and (**e**) binding of particles. (**f**) Finally, the process produces spherical granules [187]. Reprinted with Permission from Elsevier [186].

Pozzolanic activity refers to the degree of reaction over time or the reaction rate in the existence of water between a pozzolan and Ca(OH)_2_ or Ca^2+^ [5,68,77,78]. Such property of FA can be evaluated based on its strength in line with ASTM C 311. The pozzolanic activity states the reactivity of pozzolan for a pozzolanic reaction. This activity could be measured by examining the strength of 50-mm mortar cubes without and with a pozzolan, as stated in ASTM C 311. The pozzolanic activity of SCM relies on the particle size distribution, silica content, and surface fineness of SCM [186]. Class C FAs have pozzolanic and cementitious properties [49]. Class F FAs have only pozzolanic properties because they can only hydrate with cement hydration products. Mortars containing FA generally achieve minimal strength at initial ages, considering that FA’s pozzolanic reaction is commonly smaller than the hydration of cement at the beginning [184]. FA’s pozzolanic property is also commonly motivated by forming an aluminosilicate gel between the particles of binder pastes [187,188]. Figure 12 depicts the descriptive prototypical of the granulation-alkali activation technique. Figure 12a shows the inclusion of the alkali activator to the precursor particles. In the granulation process (Figure 12b), the alkali activator moistens the particles. Then, the reactive materials are dissolved (Figure 12c) and form an aluminosilicate gel (Figure 12d), motivating binding the particles together (Figure 12e). Finally, the process produces spherical granules (Figure 12f). The silica in FA reacts with calcium hydroxide, which comes from hydrating the silicate phases, producing the calcium silicate hydrate (C-S-H) that occupies most of the microstructure space and have higher binding properties than that of portlandite [180,189].

## 8. Fresh State Properties

FA significantly affect the fresh state properties of concrete. This section presents fresh state properties of FA-based concrete, including workability, setting time, segregation, and bleeding.

### 8.1. Workability

Workability is a broad and subjective term describing the consolidation, placing, ease of mixing, and finishing of fresh concrete with minimal loss of homogeneity [77]. Such property is affected by water content, w/c ratio, mix proportions, size, shape, grading, and surface texture of aggregates [78]. According to previous studies, the spherical shape of FA and the packing effect of classified FA particles reduce the water needed to achieve the desired workability [78]. In general, selecting the ash or removing the coarsest fractions from within “run-of-station” FAs offers many benefits, such as increased pozzolanic activity, rapid strength gain, and low water demand. The spherical shape of FA boosts fresh concrete’s workability, while its small size particles enable it to play as a voids filler; thereby, resulting in durable and dense concrete. It is reported that using spherical-shape FA particles resulted in high packing density, low water retention, and reduced water demand for a desired workability [190]. Commercially, the FA dosage is restricted to 20% by entire cementitious material mass, with still affecting the concrete’s workability positively [96]. The use of FA at 25% or 30% is helpful for workability, but it negatively influences the concrete strength [191,192]. Furthermore, replacing at least 50% of cement with FA results in decreased workability [108,193]. 25–30% of FA is recommended for concrete when there are concerns for sulfate attack, alkali-silica expansion, or thermal cracking [194]. It was reported that when a concrete mix design contained not more than 15% of mineral admixture (180 kg/m^3^), FA (180 kg/m^3^), w/c (0.42), and GGBS (80 kg/m^3^), the concrete had superb workability, durability, and mechanical properties [194]. Furthermore, FA can be used in fiber-reinforced concrete mix to overcome the reduction in workability because of the use of fibers [16].

### 8.2. Setting Time

As a construction material, concrete’s practical use depends on its plasticity in the freshly mixed state and subsequently hardening with considerable strength [77,78]. The initial and final setting times of concrete can be measured using ASTM C 150. Generally, the final setting could be attained at ambient temperature in no more than 2 h. Class C FAs with calcium-content additives (CaCl_2_) and large CaO content reduce the setting time of FA-based AAM paste. In geopolymerization, Al^3+^ or Si^4+^ retort with Ca^2+^ either from the exterior Ca additive content or in the FA to shape calcium aluminum silicate ((C-A-S-H) H = H_2_O, A = Al_2_O_3,_ S = SiO_2_, C = CaO) gel, C-S-H gel or calcium aluminate hydrate (C-A-H) gel in the attendance of water [28,29,195]. Ca^2+^ is advantageous in hastening the nuclei creation and accumulation of the C-S-H and C-A-S-H gels. The fast creation of amorphous C-S-H and C-A-S-H gels contributes to reduce porosity and minimize the setting time of the products, whereas a fast setting time adversely influences the creation of the AAM gel (N-A-S-H) [123]. A larger concentration of NaOH could extend the time required for setting by restraining calcium leakage and permitting the normal geopolymerization method to govern the AAM paste setting [196]. Furthermore, FA’s small reactivity extends the setting time of FA-based AAMs. Thus, curing is an essential step; that is, AAM pastes must be reserved within a sensible moisture and temperature range. Extending curing time endorses creating a cross-linked binding and a dense microstructure [133,197] as curing temperatures rise from 30 to 50 °C, the reactivity of FA increases. Furthermore, when curing temperatures are limited within the range of 60 and 90 °C, the geopolymerization is near complete [198].

### 8.3. Segregation and Bleeding

Bleeding in concrete is a type of segregation categorized by the rise of a specific amount of water to the surface of the newly positioned material [77,78]. The availability of an appropriate amount of very fine aggregate (<150 µm) reduces bleeding [5,199] given the incapability of the solid concrete to retain all the water mixed from preparing concrete and during the process of material downward settling [85,97]. Concrete bleeding can be tested through the ASTM C 232 standard test method [200]. It was reported that including FAs improves the concrete workability by diminishing segregation and bleeding [201]. Another reason is that dense concretes have tight and smooth surfaces with minimal bleeding [202,203].

**Figure 13:**
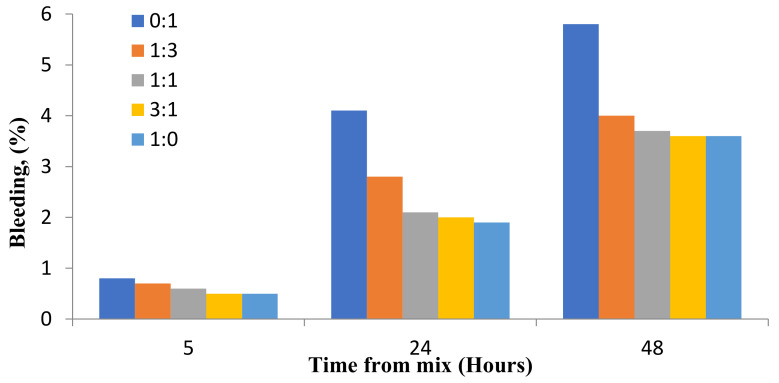
Effect of FA on water bleeding [208]. (Modified with improvement from [207]).

Increasing FA decreases the bleeding rate due to the decreased permeability, increased cohesiveness, and decreased water content of concrete [157,204,205]. It was documented that substituting 35–50% of cement with FA reduced the water need for obtaining the required slump of 5–7% in mortars [146]. The rate of bleeding of the FA’s suspension in water is compared with that of OPC in water in Figure 13, with both suspensions containing equal weights of particles and fluid [206,207]. The rate of bleeding is low for FA considering its low specific gravity and fine graded particles, thereby resulting in settling and the ability to attract and retain water on the particle surface [5].

## 9. Mechanical Properties

After settling, the concrete must be strong enough to withstand the applied structural and service loads. This section presents the mechanical properties of FA-based concrete including compressive strength, splitting tensile and flexural strength, and modulus of elasticity. Table 9 summarizes the effect of FA on different mechanical properties of FA-based concrete.

### 9.1. Compressive Strength

Table 10 presents the field measurement data of concrete produced with OPC and FA. The strength of FA-based AAM depends on curing condition, Si/Al ratio, alkali solution, calcium content, and various additives [5,216,217]. As FA initially reacts with liquid at a reasonably slow rate, the compressive strength of the concrete at the first few days after mixing is low; however, high strength is developed at the longer ages [218,219]. The size of the FA particles is important for the strength development of concrete. In long periods, the strength increases with FA up to a replacement rate of about 25–35%, after which the strength starts to decrease with more added FA. Generally, class C FA is used within 15–40% of the total binder of concrete. Class C FAs improves the strength if the replacement level is restricted to 25% by the mass of the binder.

Table 11 presents field measurement data of compressive strength of FA-based AAM. A strong correlation exists between calcium content and strength enhancement [223]. Furthermore, the pozzolanic reaction, packing effect, and hydration rate affect the compressive characteristics of mortars with FA. As was reported, the packing effect on FA mortar’s strength at early ages is larger than that induced by the pozzolanic reaction [152]. The combination of NaOH with Na_2_SiO_3_ and KOH with potassium silicate (K_2_SiO_3_) as alkaline liquids has been studied in previous research [118]. They discovered that the form of the alkaline liquid has a significant effect on the mechanical strength of concrete. The mixture of NaOH with Na_2_SiO_3_ exhibits a higher concrete compressive strength than that of KOH with K_2_SiO_3_. FAs with a higher amount of CaO produce a higher compressive strength, particularly in the early ages [40,224]. According to an experimental study that involved the geopolymerization of 16 natural Si-Al minerals, the percentage of potassium oxide and calcium oxide in the raw material, as well as the molar ratio of Si-to-Al, the liquid alkali form, the degree of Si dissolution, and the molar ratio of Si-to-Al in a solution, have a significant impact on the compressive strength of AAMs [225,226]. Furthermore, an increase in the Si/Al ratios typically enhances the compressive strength of FA-based AAM.

The fineness of FA has also a significant influence on the strength development of concrete [152,238]. Moreover, different treatments, such as magnetic extraction, sieving, mechanical separation, and grinding, can be adopted to alter FA’s properties to amend the compressive strength of FA mortars [239]. Furthermore, the presence of calcium in FA or its usage as an admixture is valuable for making the amorphous C-A-S-H and C-S-H gels and reducing the porosity to improve the compressive strength of AAM. An investigation discovered that the compressive strength of cement pastes and mortars containing FA improved by curing at high temperatures [239,240]. It was also reported that relative humidity significantly affected the strength development [239,240]. Curing considerably influences the compressive strength of FA-based AAM by varying the density and porosity of the AAM [133].

As was reported, AAM containing NaOH solution (0.5, 1, and 1.5%) with different FA/slag ratios (0, 20, 40, and 60%) cured for 1, 3, 7, and 28 days exhibited an improved compressive strength with the inclusion of slag [189]. The optimum compressive strength was 93 MPa [189]. In a previous study [241], recycled coarse aggregate (RCA) obtained from crushed structural concrete beams and clay was utilized in FA-based AAM to produce concrete with adequate mechanical characteristics [197,241,242,243]. It was reported that AAM with RCA developed a less compressive strengths of up to 10.3 MPa compared to that with natural coarse aggregates [241]. However, the compressive strength was still within the classic strength distribution. Additionally, the total void ratio of AAM with RCA (21.7% to 26.9%) was comparable to that with natural aggregates (24.2% to 27.4%), and the water penetrability values were 0.71 to 1.47 cm/s against 1.18 to 1.71 cm/s [241]. In another study, RCAs from existing concrete with a compressive strength of 30 to 45 MPa and larger calcium FA content was used to create an FA-based AAM [244]. They reported that the FA-based AAM with RCA displayed compressive strengths of 30.6 to 38.4 MPa, which was somewhat smaller than that of FA-based AAMs with crushed limestone [244].

### 9.2. Splitting Tensile and Flexural Strength

As well as its brittle nature, concrete is well known for its weakness in tension. The splitting tensile strength of FA-based AAMs can be enhanced using additives, such as sweet sorghum fibers and polyvinyl alcohol (PVA) fibers together with N-carboxymethyl chitosan [133]. A group of researchers made AAM with FA and alkali-pretreated sweet sorghum fibers that were acquired from the wastage of bagasse after removing the juice from the sweet sorghum stubbles for ethanol production [245]. They reported that when the percentage of sweet sorghum fibers was 2%, the tensile strength of the AAM increased by almost 36% [245]. Adding fibers to the mixture by a certain percentage limited the increase of micro-cracks, improving the tensile strength. However, the additional increase in fiber content led to the fiber agglomeration, resulting in a rise in air foams tricked in the mixture and non-uniform fiber distribution, and decreased tensile strength. Adding cotton to an FA-based AAM exhibited a similar trend for tensile strength [246]. It was reported that the increase in the tensile strength of chitosan- and fiber-reinforced FA-based AAM was mainly due to macro-and micro-fibers that could improve hydrogen bonds, load transfer, and fiber bridge action, and reduce the micro-cracks growth and expansion.

The flexural strength of FA-based concrete is considerably improved by integrating various kinds of short artificial fibers, such as polypropylene and PVA, through a linking influence during the macro-and micro-cracking of the AAM matrix under bending. The fibers for strengthening FA-based AAM composites include PVA fiber [247], steel fiber [2,248], sweet sorghum fiber [245], and cotton fiber [249,250]. FA-based AAMs can undergo stiff failure with small tensile strength and fracture toughness [133]. To obtain a great flexural strength, 2% of PVA fiber, 2% of steel fiber, and a hybrid combination of 1% of PVA and 1% of steel fiber were added. Besides, some studies showed the deflection toughening performance of the hybrid fiber-reinforced FA and reported that the flexural and bond strengths between the AAM matrix and PVA fiber were greater than those with cement paste matrix. The AAM matrix’s alkalinity shows no influence in the degradation of steel and PVA fibers [133]. Furthermore, cotton fabric layers were also included in the FA-based AAM composite to increase the flexural strength of the composite [249,250]. The improved flexural strength was exhibited in FA-based AAM in the range of 8.2 and 31.7 MPa, while the cotton fiber content was raised from 0 to 8.3%. Furthermore, FA’s flexural strength is disturbed by the alignment of cotton fabric layers [133]. The great flexural strength of FA-based concrete reinforced with cotton fabric placed horizontally can be accredited to the enhanced load dispersal consistency within the successive cotton fabric layers. The AAM with a vertical fabric alignment endured delamination and detachments between the AAM matrix and the cotton fabric, and had a less flexural strength [250].

### 9.3. Modulus of Elasticity

It has been demonstrated that the modulus of elasticity and compressive strength of FA concrete are strongly correlated [228,229]. Generally, using FA in concrete increases the elastic modulus of concrete given the FA’s pozzolanic action over the concrete’ hydration period [205]. The low modulus of FA-based concrete is attributed to the low early strength development of the concrete [213]. AAMs with the average density of 2350 kg/m^3^ have a higher elastic modulus than those containing OPC [251].

**Figure 14:**
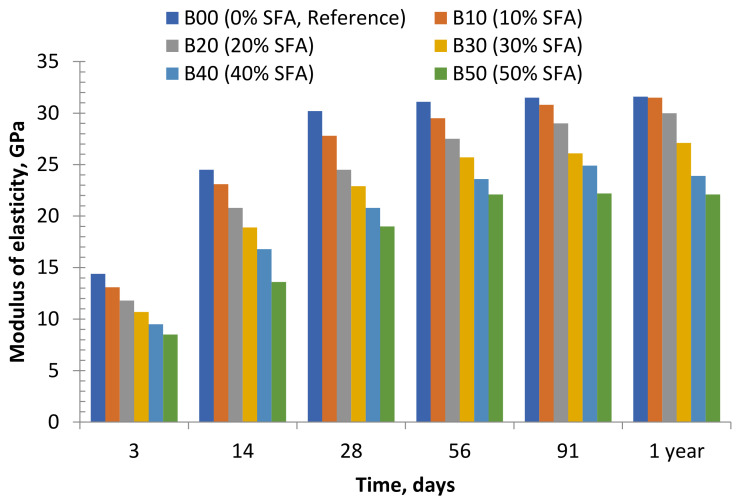
Modulus of elasticity versus age of Saudi FA (SFA)-based concrete [41]. Reprinted with permission from Elsevier [41].

Long-term behavior is a significant feature of the durability of FA-based AAM composites. The long-term strength and elastic modulus of concrete increase with an increase in FA up to a replacement rate of approximately 25–35%, after which the strength decreases with the further addition of FA [252]. Furthermore, the elastic modulus of FA concretes with the ranges from 10% to 50% fine aggregate replacements was more than that of the control mixture at different times, indicating that elastic modulus increases with the FA content and age. Figure 14 shows the relationship between elastic modulus of Saudi FA (SFA)-based concrete with time [41]. This tendency is apparent between 40% and 50% replacement levels, but a maximum strength at all times was obtained at 50% fine aggregate replacement.

Furthermore, incorporating FA has a higher impact on elastic modulus than on compressive strength [253,254]. This phenomenon can be attributed to the various distributions of the C-S-H particles and their disposition regarding the other phases and the unreacted FA. Similarly, the combination of condensed graphene oxide (CGO) into AAM was considered. Furthermore, in the spectra of AAM, the spectral absorbance associated with silica-type cross-bridging was improved. Their SEM image revealed that CGO changed the AAM’s morphology from a permeable to a pore-filled composite. The greatest elastic modulus with 376% increase was reached after adding 0.35% CGO.

## 10. Heat of Hydration

The heat evolution upon complete hydration of a particular amount of unhydrated cement at a given temperature is a property of FA-based concrete (Table 12) [5]. The total volume of free heat and the quantities of heat delivered by a single hydrating compound can be considered as reactivity indicators. Besides, heat of hydration exemplifies the settling and toughening behavior of cement pastes and forecasts the temperature increment [77,78]. The concrete temperature due to hydration is mainly governed by the mix and material properties and ecological factors [78,255]. In terms of using FA, FA affects cement hydration rate, as indicated by the hydration concept. An opposing effect of FAs on hydration kinetics was observed because of differences in their chemical composition. For instance, the effect of Class C FA on hydration is variable, while Class F FAs decrease the hydration. Previous research has revealed that hydrating clinker phases were improved when FA is available within the first hydration days [256,257].

Furthermore, FA particles exhibit a similar phenomenon to the glass. It was illustrated that glass particles were shielded with fibrous hydrates layer, signifying C-S-H’s propensity to nucleate on glass surfaces. In addition, the C-S-H formed from hydrating FA cement pastes is comparable to the cement substitution by metakaolin and slag, with a high surface area and a foil-like morphology [258]. The CaO/SiO_2_ ratio of the C-S-H produced in mixes containing FA was less than those in mixes with cement [68].

**Figure 15:**
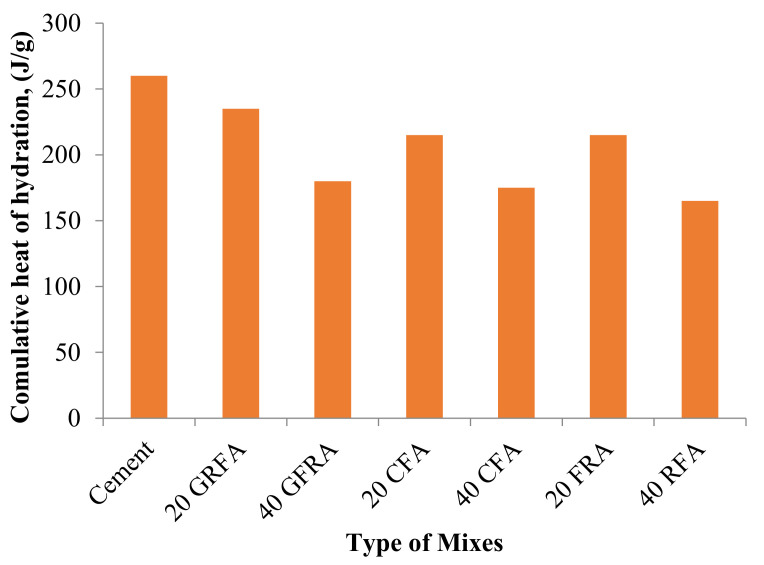
Cumulative heat of hydration after 2-days for cement and different FA-based pastes [140]. Reprinted with permission from Elsevier [140].

The total heat produced by hydration of OPC and mixed pastes with FA in 2 days is shown in Figure 15 [130]. As shown in the figure, the total heat emitted during the hydration of mixed pastes was lower than that of OPC paste. With an increase in the amount of FA, the amount of hydration heat decreased, which was attributed to the combined impact of the increased FA content and diluting OPC [259].

**Table 12:** Summary of Ca-based and Na/K-based activators in FA concrete.

Mix	Activator	Records	Findings	Refs.
FA + OPC	Ca(OH)_2_	Mild activation with pH between 7 and 13. Enhanced pozzolanic activity in long-term behavior.	Making reaction with soluble salts to produce insoluble Ca-compounds and increase the alkalinity.	[159]
FA		Hydrothermal treatment at a temperature of 130 °C.	Helped the formation of Al-substituted 11 Å tobermorite and hibschite.	[260]
FA + OPC	CaO	For low-Ca FA, CaO was simply beneficial throughout early ages. As for high-Ca FA, the CaO was beneficial during both early and later ages.	Optimum dosage of 3% CaO. No enhancement influence was found with CaO content more than 5%.	[261]
FA		CaO was as a less effective additive compared to Ca(OH)_2_.	CaO displayed favorable effects when AAM cured at ambient temperature exhibited unfavorable influences when cured at elevated temperatures.	[262]
FA + Lime	CaCl_2_	It lowered the pH of pastes, however, enhanced Ca(OH)_2_ dissolution.	4% CaCl_2_ at 23 °C reduced early strength and improved later strength, but it improved both from 35 to 65 °C.	[263]
FA + Limestone		Mixture with 1.7% CaCl_2_ and 10% FA is the optimum mix.	CaCl_2_ offered a considerable improvement in both early and long age strength and in accelerated setting time.	[248]
FA + Lime	CaSO_4_	Encouraged the formation of ettringite and dihydrate calcium sulfate.	Accelerated the pozzolanic activity of FA and considerably enhanced the early age strength of the binder.	[264]
FA + OPC		Anhydrite is more efficient at amending early age strength, however, it is less efficacious at enhancing later age strength than gypsum.	10% anhydrite improved the 3 days *f_c_* by 70% and showed lower porosity and smaller pore sizes.	[265]
FA	NaOH	Growing T triggered a reduction of Si/Al in aluminosilicate gel.	Hydrates including traces of zeolite plus amorphous alkali aluminosilicate.	[266]
FA		Cured for 24 h at 30 °C.	at high (OH/Al) ratio, NaOH promoted more 6-coordinate Al.	
FA + Slag		Curing at ambient temperature.	At 28 days, *f_c_* = 50 MPa with 10 M NaOH.	
FA + OPC	Na_2_CO_3_	3% and curing in ambient temperature.	Mortar exhibited 28 days *f_c_* = 14.8 MPa (*f_c_* = 22.0 MPa when there was no activator group).	
FA + Ca(OH)_2_		Na_2_CO_3_ did not amend strength for NaOH-activated FA.	A noticeable enhancement in microstructure and strength was attained.	[267]
FA + OPC	Na_2_SO_4_	Ash can be activated at earlier ages by increasing the creation of A_Ft_ and alkalinity.	Compressive strength of mortar is improved by 40% for the first 3–7 days.	[105]
FA + Lime		Na_2_SO_4_ enhanced lime consumption on the first day and then did not thereafter.	4% NaSO_4_ improved paste strength at both earlier and later age.	[248]
FA + NaOH		Cured at 85 °C.	Converting of N-A-S-H gel into zeolites is enhanced. Sulfates are acting as an activation retarding agent once NaOH is the activator.	[268]
FA + OPC	K_2_SO_4_	1% K_2_SO_4_ and cured at 20 °C.	It is beneficial in lowering the total porosity and improving the early strength.	[269]
FA	Na_2_SiO_3_	Modulus was kept maintained at 1.0 when it was cured at 80 °C.	Activation of Na_2_SiO_3_ is not appropriate for high-Ca ash, however appropriate for high-Ca ash.	
FA		Cured at 60 °C for 24 h.	The strength of paste was largely linked to the gel-like hydrates at modulus of 1.64, and the formation of crystalline Na_2_SiO_3_ resulted in higher compressive strengths with the corresponding modulus = 1.0.	
	
FA + NaOH	Na_2_CO_3_	The major cause of strength was not due to a high pH at the early stage of NaOH formation.	Na_2_CO_3_ did not amend the compressive strength of the NaOH-activated FA binder.	
FA + OPC		Secondary phases, such as AFm and gaylussite, were preferred.	Na_2_CO_3_ favored precipitation of C-A-S-H-like gel over (N, C)-A-S-H-like gel.	

It was also reported that the decrease in the total heat evolution with incorporating FA can be because of the relatively low specific surface area and low solubility of the aluminosilicate present in FA [270].

## 11. Utilizations of FA

**Figure 16:**
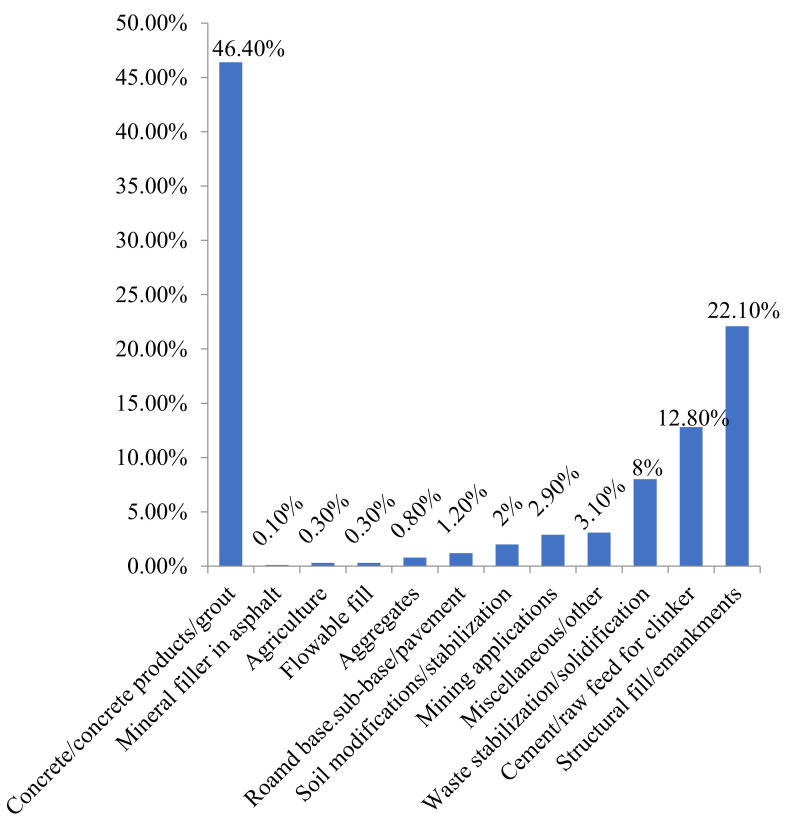
Common applications of FA around the world [273]. Reprinted with permission from Elsevier [272].

The construction industry is one of the world’s fastest-growing sectors. According to statistics, approximately 260 billion tons of cement is needed to meet various global construction needs [243]. Over the next ten years, it is estimated that this quantity will increase by 25%. Using waste material in concrete is one way to minimize cement demand. Eco-friendly concrete can be made from various waste materials, including FA as a partial cement substitute. FA disposal and utilization has been a big concern given the reliance of many countries on thermal power generation. Scientists, technologists, and engineers will face a new challenge in the future management of FA. Furthermore, FAs as binders or fillers are usually dumped in landfill and open fields, creating health hazards and environmental pollution issues [16,271]. Many authors have evaluated its potential as a building and construction material due to its abundance and excellent pozzolanic properties. For potential housing projects, FA is a promising partial cement replacement material [272]. Generally, FA is used as an SCM in concrete production and other materials shown in Figure 14. Several FA utilization areas include mine filling, construction of roads, and several building components (e.g., bricks and tiles) (Figure 16).

**Figure 17:**
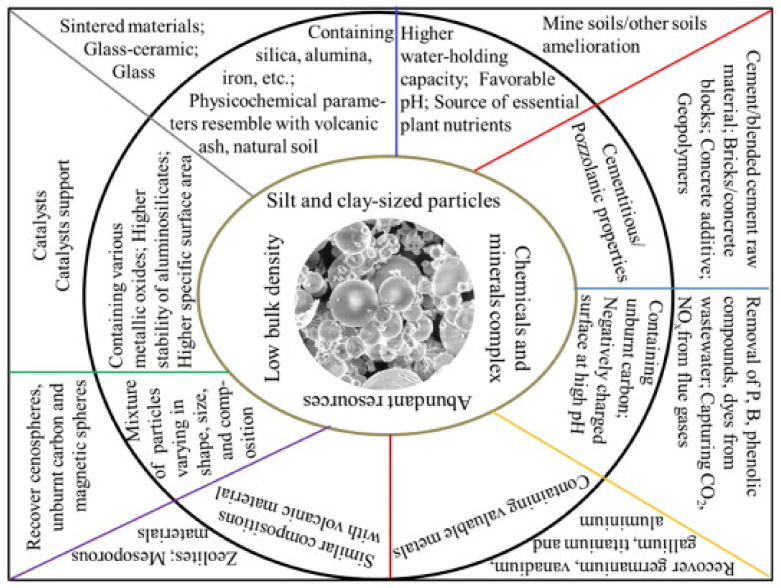
Typical applications of FA [92]. Reprinted with permission from Elsevier [92].

Researchers have identified key applications for FA in the future based on the yearly time series data by recognizing a pattern of development using regression analysis [274,275] and the amount of FA to be used in choice and specific applications have been predicted, as shown in Figure 17 [92,276]. The greatest use of ash, to the amount of 44.19%, was anticipated in the concrete and cement industries in 2020–2021. The next-highest, 15.25% of ash, is to be used in ash dyke raising, roads, and embankments, another 12.49% in landfilling and retrieval of lowland areas, 7.61% in bricks and blocks, 8.84% in tiles and mine-filling, 2.47% in cultivation, and 9.14% in other structures. Therefore, the top five application industries are (i) concrete and cement, (ii) embankments, ash dykes, and rising roads, (iii) lowland area retrieval and landfilling, (iv) bricks and blocks, and (v) mine-filling and tiles, based on the upcoming skills. The amount of FA used in other applications could change in the coming years. Based on the literature review, no study has been conducted on utilizing boiler ash in the production of AAMs. Therefore, future work should be conducted to use boiler ash as an AAM material.

## 12. Conclusions

The use of FA as an SCM in concrete could resolve the disposal and health issues induced by the generation of ash due to coal combustion in industrial boilers or electric utility. The use of FA in concrete could also help lower the pollution induced by the cement factories by decreasing the CO_2_ emissions in the cement production. FA-based concrete exhibit high pozzolanic activity, opposed to their mineralogical characteristics and fineness properties. Most of the existing studies did not consider the effect of main influential parameters, particularly particle size distribution, particle packing effect, and alkaline activator solutions, on the strength development of FA-based concrete. FA is potentially utilized to substitute a significant volume of OPC (up to 50%) without influencing concrete durability. However, using 50% FA as a replacement by the cement weight may negatively affect the concrete strength. FA’s fineness has a significant role in concrete because it affects the workability and the rate of pozzolanic activity in concrete, thereby contributing to the enhancement of the properties of concrete. FA-based concrete demonstrated a superior performance to that of OPC-based concrete in resisting sulfate attack, acid attack, and carbonation. To approve the favorable influences of FA on concrete characteristics and durability problems, the following research directions are recommended for future studies.

– The manufacturing and improvement of the performance of FA-based AAM must be controlled, and the reaction aspects of the material should be studied in detail. To this end, several facets, for instance, kinetics, thermodynamics, sympathies of intermediates and perceptions into their systems, and the grades to which the Si-O-Al are polymerized and oligomerized, should be studied. These will develop progressively improved performance of the concrete when the extra additives or components are involved. However, further research is needed to confirm that the manufacturing–structure–behaviors correspondence is accurate.
– The majority of FA-based AAMs are stiff and susceptible to cracking. This performance obliges restrictions in applications and influences the long-term durability of AAMs. Therefore, innovations in the preparation must be applied to produce improved FA-based AAM composites.
– Currently, FA-based AAMs are only formed at the research laboratory scale with empirical formulations. Thus, several studies on FA-based AAM production are required and must endeavor to adopt FA-based AAMs on a large scale.
– The performance of FA-based AAMs for immobilization, toxic metal adsorption and the sealing of CO_2_ remained unsatisfactory. However, shifting the guidelines for preparation is worthy of further investigation.
– As an alternative material to conventional concrete, FA-based AAM may be endowed with unique properties or additional functionalities. Therefore, novel applications of FA-based AAMs are worth discovering. For example, FA-based AAMs with biomass can be approved as new light-weight and incombustible materials.
– The potential use of FA in producing high-strength and self-consolidating concretes must be studied.
– Fibers must be used to increase the strength and longevity of FA in the concrete hardened state.
– The use of FA in the design of eco-friendly buildings and cities should be highlighted.

## Figures and Tables

**Table 1:** Specifications for class F and class C fly ash (FA).

Property	Specifications	Rate	Class C	Class F	Ref.
Optional chemical requirements	Fe_2_O_3_ + Al_2_O_3_ + SiO_2_	min%	50	70	[35,45,46,47,48,49,50,51]
	SiO_3_	max%	5	5	
	Moisture Content		3	3	
	LOI		5	5	
	Available alkalis		1.5	1.5	
	Pozzolanic activity/cement (7 days)		75	75	
	Pozzolanic activity/cement (28 days)		75	75	
Optional physical requirements	Fineness (+325 Mesh)	min%	34	34	
	Water requirement	max%	105	105	
	Autoclave expansion		0.8	0.8	
	Uniform requirements^2^: Fineness		5	5	
	Uniform requirements^2^: Density		5	5	
Optional physical requirements	Cement/Alkali Reaction: Mortar expansion (14 days)		--	0.020	
	Multiple factors (LOI _x_ fineness)		--	255	
	Uniformity requirements: Air entraining agent		20	20	
	Increase in drying shrinkage		0.03	0.03	

**Table 2:** Common properties of FA.

Parameters	Range	Refs.
Uniformity coefficient	3.1–10.7	
Permeability (cm/s)	8 × 10^−6^–7 × 10^−4^	[52]
Compression index C_c_	0.05–0.4	
Consolidation coefficient C_v_ (cm^2^/s)	1.75 × 10^−5^–2.01 × 10^−3^	
Specific gravity	1.90–2.55	[23]
Internal friction angle (j)	300°–400°	
Cohesion (kN/m^2^) and Plasticity	Negligible and non-plastic	
Maximum dry density (g/cc)	0.9–1.6	[31]
Optimum moisture content (%)	38.0–18.0	

**Table 3:** Distribution of characteristics of different oxides production technique of FA.

Category	Oxide	Diffusion	Effective Agent	Ref.
Network formers	Fe_3_O_4_	– Usually available in Magnetite and surface of soluble vitreous. – Fe-distribution does not exhibit any particular correlation to Si and Al. – Iron-rich FA particles are mostly solid and spherical.	Aluminum oxide.	[57,58,59]
	Fe_2_O_3_	– Concentrated in the exterior hull.	The boiling degree of silica is unlike iron oxide.	[60]
	Al_2_O_3_	– Al/Si ratio in all density fractions of high-Ca FA is of the same order of magnitude. – Enriched in the cenosphere. – Poor FA spheres’ outer layer. – Very small percentage is available as 6-coordinate in mullite which does not react. – Mainly in 4-coordinate associated with silicon in FA glass.	Aluminum oxide and silica have a similar boiling point of 2980 °C and 2950 °C, respectively.	[39,57,61,62]
	SiO_2_	– SiO_2_ is not reactive in the crystalline quartz and mullite form. – Poor in FA spheres’ outer layer. – Mainly available as FA glassy contents revealed 4 percent of silicate is available in quartz form.	Silica’s crystalline degree is chiefly controlled by the coal type, cooling process, and combustion temperature.	[39,57,58,63]
Network Modifiers	MgO Na_2_O K_2_O	– Vary largely in different density fractions of FA. – Rich in water-soluble contents. – Associated with total glass content. – Concentrated in the exterior hull.	The boiling points that are the same in these components are smaller than silica and aluminum oxide, which produces a more volatile concentration of constituents in the outer layer.	[39,61,62]
	CaO	– The maximum CaO content limited in the glassy particles could be near to anorthite (<25 wt. %). – Rich CaO in water-soluble contents, including free and anhydrate lime. – Rich in terms of fine particle size ranges and focused in the exterior hull. – CaO content is large regarding phosphorus or sulfur, which is ascribed to the calcium oxide bearing minerals decomposition including gypsum in coal.	The calcium oxide mineral distribution will be controlled by the presence of SO_3_ and free lime.	[60,61,62]

**Table 4:** Global production and consumption of FA.

Type	Million Tons	Year	Ref.
Larger producer	India (112/per year)	2019	[75,76]
Consumed	3840	2015	
	4032	2032	
Fly ash market	US$4.13 billion and US$6.86 billion in 2018 and 2026, respectively.		

**Table 7:** Comparison of measurements in standards for the properties of pozzolanic materials.

Property	EN 13263	ASTM C 1240
Reference mix	225 g of distilled water 1350 g of standard sand 450 g of test cement	242 g of distilled water 1375 g of standard sand 500 g of test cement and X g of Flow agent (superplasticizer)
Pozzolanic (by replacement)	45 g	50 g
Superplasticizer (Flowability determined using specific equipment)	Superplasticizer is inappropriate with EN-934-2. As much superplasticizer as necessary to determine the standard flow (±5 mm).	Dry high range water reducer in conformity with C494 Type F. Adding superplasticizer to gain a flow mixture of 100–115% (summation of 4 measurements which done with a special caliper).
Curing (after 24 h in the mold)	Submerged in 20 ± 1 °C water temperature for 27 days.	In airtight glass containers at temperature of 65 ± 2 °C for 6 days.

**Table 8:** Review on grain size and grading quality of FA.

Class of FA	Year	Grading Quality	Curvature Coefficient	Uniformity Coefficient	Notes	Ref.
F	1990	Low	1.56	4	Used for foundations of buildings and roads	[160]
			-	3		
				2.4		
				2.8		
		High	1.82	9		
	2001	Low	0.95	2.14	About 70% of FA are made of particulate matter with a diameter of 2–60 μm (size of silt), 25% with diameter 60–200 μm (size of fine sand) and 5% with medium-sized sand (200–600 μm).	[161]
			0.76	3.67	In general, the FA particles have a size equivalent to that of the sludge, the Gulbarga FA being better than the others. Neyveli FA and Vijayawada FA are very similar in size.	[162]
			0.95	2.14		
			0.74	6.67		
	2003		1.01	4.82	FA can be classified as a non-plastic ML-type sludge, following the unified soil classification system.	[163]
			0.9	5.65		
C	2004	High	1.03	11.2	The particle size analysis was conducted using hydrometer and sieving methods (ASTM D 422, D 1140). The distribution curve of grain size indicates most sludge size uniform material.	[164]
		Low	1.04	3.16	Indian coal FAs consist predominantly of silt-size fraction and some clay-size fraction.	[165]
			2.47	5.5		
		High	1.14	6		
		Low	1.09	1.59		
			0.61	5.7		
F	2005	High	1.68	50	Original FA with 31% (average size of 19.1 μm) was retained on No. 325 sieve (45 μm). All classified FA (average size of 6.4 μm) passed through No. 325 sieve.	[166]
			2.39	22		
			1.01	10.3	-	[167]
			2.98	36.5		
	2007	Low	3.21	3.67	C_u_ and C_c_ values were mentioned as per Indian Standard Procedure.	[168]
		High	1.96	7	FA has particles the size of clay (5%), sand (17%), and silt (68%).	[169]
	2010	Low	0.94	4.02	The particle size analysis was carried out by wet dispersion method in water using a Malvern 3601 particle size analyzer.	[170]
			0.93	3.96		
			0.91	4		
	2011		18.15	30	-	[171]
			26.42	28		
	2012		0.67	16.67	The distribution of particle size was attained from laser granulometry.	[172]
C	2013	High	1.2	12.5	-	[173]
		Low	0.91	6		
		High	1.05	18.8		
			1.08	13.8		
F	2014	Low	1.8	7.5	FA was 85.4% finer than a No. 200 sieve (0.075 mm diameter)	[174]
	2016		1.12	2.13	-	[175]
	2018		1.55	5.88	86.6% FA passed 75 μm sieve	[176]
	2019		3.12	5.44	-	[177]

**Table 9:** Summary of the effect of FA on mechanical properties of concrete.

Properties	Influence of FA	Ref.
Hydration chemistry	High-Ca FA: exhibits concurrent cementitious and pozzolanic reactions and gaining high early strength from the following reactions. 2S + 3CH → C_3_S_2_H_3_ C_3_A + CSH_2_ + 10 H → C_4_ASH_12_ A + CSH_2_ + 3CH + 7 H → C_4_ASH_12_ A + 4CH + 9 C + H → CH H → C_4_AH_13_ Low-Ca FA: exhibits mostly pozzolanic reactions. 3CH + CSH_2_ + A + 7H → C_4_ASH_12_ 3CH + 2S → C_3_S2H_3_ A + 4CH + 9 H → C_4_AH_13_	[23,32,130,209,210]
Abrasion	Abrasion resistance is mainly correlated with the compressive strength of FA concrete and there is not a clear association to the addition of FA.	[211]
Splitting tensile strength (*f_t_*)	FA at 50% substitution in enhanced concrete *f_t_* by 20%; however, when the substitution rose to 70%, a 35% reduction was observed compared to OPC concrete.	[212]
Flexural strength (*f_bt_*)	FA concretes with less than 50% replacement level showed greater *f_bt_* than OPC concretes. With FA at substitution levels of 40% to 80%, the *f_bt_* of FA concret reduced marginally with increased FA content.	[213,214]
Compressive strength (*f_c_*)	FA typically lowers the initial-age *f_c_* of concrete. This strength deficiency will diminish given the pozzolanic reaction at later ages.	[215]

**Table 10:** Review on field measurement data of concrete produced with cement and FA.

Structural Element	Age (Days)	Exposure/ Service Situation	Concrete		Compressive Strength (MPa)		Cover (mm)	Carbonation		Footnotes	Ref.
								Depth (mm)	Rate (mm/Year)		
			OPC:FA	w/c	Design	In-Situ					
Slab-on-grade	28	Industrial	100:0 75:15 80:20	0·65 0·60 0·60	20 20 20	Cube: 57·0 Cube: 49·0 Cube: 57·0	80 60 60	7·0 1·0 4·0	1·3 0·8 0·2	Slight corrosion found in FA concrete	[220,221]
Foundation	25	-	100:0	0·52	21	Cube: 66·5	-	-	-		[222]
Dam monolith	25	Base 5 ft (1·5 m) above high	80:20 100:0 80:20	0·52 0·52 0·52	21 - -	Cube:69·0 Core: 36·5 Core: 28·0	-	0 5·0 23·0	- 1·0 4·6		[146]
Outfall canal– Wall	20	water level -	100:0 80:0	0·60 0·60	- -	Core: 50·5 Core: 38·5	100 100	4.0 16.0	0·9 1·6	Insignificant cracks in both concretes; no corrosion	[92]
Bridge- embankment	10	Sheltered	100:0 75:25	0·55 0·48	30 30	Cube: 64·0 Cube: 81·5	-	1·1 0·1	0·30 0·03	Calcium hydroxide of FA concrete considerably lower than PC concrete	
Bridge—leaf pier	10	Sheltered	100:0 75:25	0·55 0·48	30 30	Cube: 47·0 Cube: 70·5	-	2·9 2·5	0·9 0·8		
Buttress dam	30	-	100:0 80:20	0·64 0·60	- -	Core: 42·5 Core: 48·0	-	5·0 8·5	0·9 1·6		[201]
Sea wall (land-ward side)	30	-	100:0 75:25	n/a n/a	- -	Core: 59·0 Core: 63·5	-	0·5 1·5	0·1 0·3		
Foundation block	33	Interiorly exposed, warm and dry	100:0 80:20	0·58 0·58	- -	- Core: 41·0	-	19·5 22·5	3·4 3·9	-	[201]

**Table 11:** Summary of compressive strength of FA-based alkali-activated material (AAM).

Material/Alkaline Activators	Compressive Strength (MPa)	Curing Time/Temperature (°C)	Mixing Temperature (°C)	Ref.
Class F FA + crushed granite stone + superplasticizer/ Na_2_SiO_3_ + NaOH 5/2	40.9–53.1	48 h; 1, 3, 7 days/ 70	-	[32,227]
FA + Crushed granite rock + river sand/Na_2_SiO_3_ + NaOH	42.0–58.0	6–72 h/60–120	AT	[228]
Pulverized coal combustion FA + Bottom ash + flue gas desulfurization gypsum/Na_2_SiO_3_ + NaOH	25.5–55.5	48 h/40	-	[229]
Class F FA/N-carboxymethyl chitosan NaOH (10 mol/L)	<30	6 days/60	AT	[230]
FA/NaOH (16.5, 14.0, 12.0, 9.5, 7.0, 4.5 mol/L)	<25.5	-/25–28	-	[122,227,231]
FA/Na_2_SiO_3_ + Na_2_SO_4_, NaOH (10 mol) CaCl_2_, CaSO_4_	26.9–32.2	48 h/65	-	[232]
FA + wastepaper sludge/Na_2_SiO_3_ + NaOH 1/5	31.2–60.6	91 days/23–60		
FA + palm oil fuel ash/Na_2_SiO_3_+NaOH	<38	24 h/65		[233]
Class F FA + Red mud/NaOH (50wt.%) + sodium trisilicate (2 mol/L)	11.3–21.3	28 days/AT	-	[234]
Class F FA + blast furnace slag/K_2_SiO_3_/Al (85 g/L) + NaOH (30 g/L)	-	7 days/RT	-	[235]
GGBS + palm oil fuel ash + FA + Manufactured-sand/Na_2_SiO_3_ + NaOH	9.0–66.0	24 h/65	-	[236]
Class F FA/NaOH 3/5	1.4–9.9	7, 28 days/60	25	[237]

AT: ambient temperature, RT: room temperature.

## Data Availability

Data sharing not applicable.

## Abbreviations

Alkali-activated material	AAM
Ambient temperature	AT
Ordinary Portland cement	OPC
Classified fly ash	CFA
Condensed graphene oxide	CGO
Electrostatic precipitator	ESP
Fly ash	FA
Fluidized-bed combustion	FBC
Ground granulated blast-furnace slag	GGBS
Loss on ignition	LOI
Pulverized coal	PC
Pollution control system	PCS
Polyvinyl alcohol	PVA
Recycled coarse aggregate	RCA
Room temperature	RT
Strength activity index	SAI
Supplementary cementitious material	SCM
Saudi fly ash	SFA
Specific gravity	SG
Submerged specific gravity	SSG

## References

[1] Baker L. (2020). World cement, 2020 vision. Altern. J..

[2] Shaikh F.U.A. (2016). Mechanical and durability properties of fly ash geopolymer concrete containing recycled coarse aggregates. Int. J. Sustain. Built Environ..

[3] Mahaboob Basha S., Bhupal Reddy C., Vasugi K. (2016). Strength behaviour of geopolymer concrete replacing fine aggregates by M- sand and E-waste. Int. J. Eng. Trends Technol..

[4] Lakshmi R., Nagan S. (2011). Utilization of waste e plastic particles in cementitious mixtures. J. Struct. Eng..

[5] Shalini A., Gurunarayanan G., Kumar R., Prakash V., Sakthivel S. (2016). Performance of Rice Husk Ash in Geopolymer Concrete. Int. J. Innov. Res. Sci. Technol..

[6] Castel A., Foster S.J. (2015). Bond strength between blended slag and Class F fly ash geopolymer concrete with steel reinforcement. Cem. Concr. Res..

[7] Madheswaran C., Gnanasundar G., Gopalakrishnan N. (2013). Effect of molarity in geopolymer concrete. Int. J. Civ. Struct. Eng..

[8] Amran Y.H.M., Alyousef R., Alabduljabbar H., El-Zeadani M. (2020). Clean production and properties of geopolymer concrete; A review. J. Clean. Prod..

[9] Amran M., Fediuk R., Murali G., Vatin N., Karelina M., Ozbakkaloglu T., Krishna R.S., Kumar S.A., Kumar D.S., Mishra J. (2021). Rice husk ash-based concrete composites: A critical review of their properties and applications. Crystals.

[10] Amran M., Murali G., Fediuk R., Vatin N., Vasilev Y., Abdelgader H. (2021). Palm oil fuel ash-based eco-efficient concrete: A critical review of the short-term properties. Materials.

[11] Abutaha F., Abdul Razak H., Kanadasan J. (2016). Effect of palm oil clinker (POC) aggregates on fresh and hardened properties of concrete. Constr. Build. Mater..

[12] Bouasria M., Khadraoui F., Benzaama M.-H., Touati K., Chateigner D., Gascoin S., Pralong V., Orberger B., Babouri L., El Mendili Y. (2021). Partial substitution of cement by the association of Ferronickel slags and Crepidula fornicata shells. J. Build. Eng..

[13] Amran M., Debbarma S., Ozbakkaloglu T. (2021). Fly ash-based eco-friendly geopolymer concrete: A critical review of the long-term durability properties. Constr. Build. Mater..

[14] Lavaniyah S., Mohammed B.S., Al-Fakih A., Wahab M.M.A., Liew M.S., Amran Y.H. (2020). Acid and Sulphate Attacks on a Rubberized Engineered Cementitious Composite Containing Graphene Oxide. Materials.

[15] Siddika A., Amin M.R., Rayhan M.A., Islam M.S., Mamun M.A.A., Alyousef R., Amran Y.H.M. (2021). Performance of sustainable green concrete incorporated with fly ash, rice husk ash, and stone dust. Acta Polytech..

[16] Gatto M., Wollni M., Qaim M. (2015). Oil palm boom and land-use dynamics in Indonesia: The role of policies and socioeconomic factors. Land Use Policy.

[17] Awalludin M.F., Sulaiman O., Hashim R., Nadhari W.N.A.W. (2015). An overview of the oil palm industry in Malaysia and its waste utilization through thermochemical conversion, specifically via liquefaction. Renew. Sustain. Energy Rev..

[18] Salim N., Nordin N.A., Hashim R., Ibrahim M., Sato M. (2012). The potential of oil palm trunk biomass as an alternative source for compressed wood. BioRes.

[19] Hwang J.P., Shim H.B., Lim S., Ann K.Y. (2013). Enhancing the durability properties of concrete containing recycled aggregate by the use of pozzolanic materials. KSCE J. Civ. Eng..

[20] Chindaprasirt P., Homwuttiwong S., Jaturapitakkul C. (2007). Strength and water permeability of concrete containing palm oil fuel ash and rice husk-bark ash. Constr. Build. Mater..

[21] Jaturapitakkul C., Kiattikomol K., Tangchirapat W., Saeting T. (2007). Evaluation of the sulfate resistance of concrete containing palm oil fuel ash. Constr. Build. Mater..

[22] Bilek V., Sucharda O., Bujdos D. (2021). Frost Resistance of Alkali-Activated Concrete—An Important Pillar of Their Sustainability. Sustainability.

[23] Gollakota A.R.K., Volli V., Shu C.M. (2019). Progressive utilisation prospects of coal fly ash: A review. Sci. Total Environ..

[24] Sata V., Jaturapitakkul C., Kiattikomol K. (2007). Influence of pozzolan from various by-product materials on mechanical properties of high-strength concrete. Constr. Build. Mater..

[25] Islam A., Alengaram U.J., Jumaat M.Z., Bashar I.I., Kabir S.M.A. (2015). Engineering properties and carbon footprint of ground granulated blast-furnace slag-palm oil fuel ash-based structural geopolymer concrete. Constr. Build. Mater..

[26] Alengaram U.J., Al Muhit B.A., bin Jumaat M.Z. (2013). Utilization of oil palm kernel shell as lightweight aggregate in concrete—A review. Constr. Build. Mater..

[27] Alengaram U.J., Al Muhit B.A., bin Jumaat M.Z., Jing M.L.Y. (2013). A comparison of the thermal conductivity of oil palm shell foamed concrete with conventional materials. Mater. Des..

[28] Sata V., Wongsa A., Chindaprasirt P. (2013). Properties of pervious geopolymer concrete using recycled aggregates. Constr. Build. Mater..

[29] Kabir S.M.A., Alengaram U.J., Jumaat M.Z., Sharmin A., Islam A. (2015). Influence of molarity and chemical composition on the development of compressive strength in POFA based geopolymer mortar. Adv. Mater. Sci. Eng..

[30] Lesovik V., Volodchenko A., Fediuk R., Amran Y.H.M., Timokhin R. (2021). Enhancing performances of clay masonry materials based on nanosize mine waste. Constr. Build. Mater..

[31] Danish A., Mosaberpanah M.A. (2020). Formation mechanism and applications of cenospheres: A review. J. Mater. Sci..

[32] Meesala C.R., Verma N.K., Kumar S. (2020). Critical review on fly-ash based geopolymer concrete. Struct. Concr..

[33] Lesovik V., Voronov V., Glagolev E., Fediuk R., Alaskhanov A., Amran Y.H.M., Murali G., Baranov A. (2020). Improving the behaviors of foam concrete through the use of composite binder. J. Build. Eng..

[34] Rukzon S., Chindaprasirt P. (2009). An Experimental Investigation of the Carbonation of Blended Portland Cement Palm Oil Fuel Ash Mortar in an Indoor Environment. Indoor Built Environ..

[35] Xie J., Kayali O. (2016). Effect of superplasticiser on workability enhancement of Class F and Class C fly ash-based geopolymers. Constr. Build. Mater..

[36] Safiuddin M., Isa M.H.M., Jumaat M.Z. (2011). Fresh properties of self-consolidating concrete incorporating palm oil fuel ash as a supplementary cementing material. Chiang Mai J. Sci..

[37] Salih M.A., Abang Ali A.A., Farzadnia N. (2014). Characterization of mechanical and microstructural properties of palm oil fuel ash geopolymer cement paste. Constr. Build. Mater..

[38] Yusuf M.O. (2015). Performance of slag blended alkaline activated palm oil fuel ash mortar in sulfate environments. Constr. Build. Mater..

[39] Posi P., Teerachanwit C., Tanutong C., Limkamoltip S., Lertnimoolchai S., Sata V., Chindaprasirt P. (2013). Lightweight geopolymer concrete containing aggregate from recycle lightweight block. Mater. Des..

[40] Chindaprasirt P., Rattanasak U., Vongvoradit P., Jenjirapanya S. (2012). Thermal treatment and utilization of Al-rich waste in high calcium fly ash geopolymeric materials. Int. J. Miner. Metall. Mater..

[41] Amran Y.H.M., Soto M.G., Alyousef R., El-Zeadani M., Alabduljabbar H., Aune V. (2020). Performance investigation of high-proportion Saudi-fly-ash-based concrete. Results Eng..

[42] Mahlia T.M., Abdulmuin M., Alamsyah T.M., Mukhlishien D. (2001). An alternative energy source from palm wastes industry for Malaysia and Indonesia. Energy Convers. Manag..

[43] Mo K.H., Alengaram U.J., Jumaat M.Z. (2015). Experimental Investigation on the Properties of Lightweight Concrete Containing Waste Oil Palm Shell Aggregate. Procedia Eng..

[44] Tangchirapat W., Saeting T., Jaturapitakkul C., Kiattikomol K., Siripanichgorn A. (2007). Use of waste ash from palm oil industry in concrete. Waste Manag..

[45] Bakharev T. (2005). Resistance of geopolymer materials to acid attack. Cem. Concr. Res..

[46] Fan F., Liu Z., Xu G., Peng H., Cai C.S. (2018). Mechanical and thermal properties of fly ash based geopolymers. Constr. Build. Mater..

[47] Kupwade-Patil K., Allouche E.N. (2013). Impact of alkali silica reaction on fly ash-based geopolymer concrete. J. Mater. Civ. Eng..

[48] Samadhi T.W., Wulandari W., Prasetyo M.I., Fernando M.R., Purbasari A. Synthesis of geopolymer from biomass-coal ash blends. Proceedings of the AIP Conference Proceedings, at the 3rd the Materials Research Society of Indonesia.

[49] Ling Y., Wang K., Li W., Shi G., Lu P. (2019). Effect of slag on the mechanical properties and bond strength of fly ash-based engineered geopolymer composites. Compos. Part B Eng..

[50] Korniejenko K., Mucsi G., Papné Halyag N., Szabó R., Mierzwiński D., Louda P. (2020). Mechanical Properties of Basalt Fiber Reinforced Fly Ash-Based Geopolymer Composites. KnE Eng..

[51] El-Chabib H., Syed A. (2013). Properties of self-consolidating concrete made with high volumes of supplementary cementitious materials. J. Mater. Civ. Eng..

[52] Mishra A. (2016). Wear Investigation of Al-SiC p—Fly Ash Composites. Int. J. Eng. Tech. Res..

[53] Ongpeng J., Gapuz E., Andres J.J.S., Prudencio D., Cuadlisan J., Tadina M., Zacarias A., Benauro D., Pabustan A. (2020). Alkali-activated binder as stabilizer in compressed earth blocks. IOP Conference Series: Materials Science and Engineering, 4th International Conference on Construction and Building Engineering & 12th Regional Conference in Civil Engineering (ICONBUILD & RCCE 2019), 20–22 August 2019, Langkawi, Malaysia.

[54] Zhang Z., Provis J.L., Reid A., Wang H. (2015). Mechanical, thermal insulation, thermal resistance and acoustic absorption properties of geopolymer foam concrete. Cem. Concr. Compos..

[55] Yan S., Sagoe-Crentsil K. (2012). Properties of wastepaper sludge in geopolymer mortars for masonry applications. J. Environ. Manag..

[56] Kanhar A.H., Chen S., Wang F. (2020). Incineration Fly Ash and Its Treatment to Possible Utilization: A Review. Energies.

[57] Chindaprasirt P., De Silva P., Hanjitsuwan S. (2014). Effect of High-Speed Mixing on Properties of High Calcium Fly Ash Geopolymer Paste. Arab. J. Sci. Eng..

[58] Chindaprasirt P., Rattanasak U., Taebuanhuad S. (2013). Resistance to acid and sulfate solutions of microwave-assisted high calcium fly ash geopolymer. Mater. Struct. Constr..

[59] Melwanki M.B., Fuh M.-R. (2008). Dispersive liquid–liquid microextraction combined with semi-automated in-syringe back extraction as a new approach for the sample preparation of ionizable organic compounds prior to liquid chromatography. J. Chromatogr. A.

[60] Abdullah N., Sulaiman F. (2013). The Oil Palm Wastes in Malaysia. Intech.

[61] Mushtaq F., Abdullah T.A.T., Mat R., Ani F.N. (2015). Optimization and characterization of bio-oil produced by microwave assisted pyrolysis of oil palm shell waste biomass with microwave absorber. Bioresour. Technol..

[62] Ahmadi R., Zainudin N., Ismail I., Mannan M.A., Abidin A.S.Z. (2016). Micro Fine Sized Palm Oil Fuel Ash Produced Using a Wind Tunnel Production System. Adv. Mater. Sci. Eng..

[63] Nuaklong P., Sata V., Chindaprasirt P. (2016). Influence of recycled aggregate on fly ash geopolymer concrete properties. J. Clean. Prod..

[64] Ali B., Qureshi L.A., Shah S.H.A., Rehman S.U., Hussain I., Iqbal M. (2020). A step towards durable, ductile and sustainable concrete: Simultaneous incorporation of recycled aggregates, glass fiber and fly ash. Constr. Build. Mater..

[65] Bashar I.I., Alengaram U.J., Jumaat M.Z., Islam A., Santhi H., Sharmin A. (2016). Engineering properties and fracture behaviour of high volume palm oil fuel ash based fibre reinforced geopolymer concrete. Constr. Build. Mater..

[66] Wang S., Baxter L., Fonseca F. (2008). Biomass fly ash in concrete: SEM, EDX and ESEM analysis. Fuel.

[67] Du H., Tan K.H. (2017). Properties of high volume glass powder concrete. Cem. Concr. Compos..

[68] Tonnayopas D., Nilrat F., Putto K., Tantiwitayawanich J. (2006). Effect of oil palm fiber fuel ash on compressive strength of hardening concrete. Renew. Energy.

[69] Chindaprasirt P., Chareerat T., Hatanaka S., Cao T. (2010). High-Strength Geopolymer Using Fine High-Calcium Fly Ash. J. Mater. Civ. Eng..

[70] Liang G., Li Y., Yang C., Zi C., Zhang Y., Hu X., Zhao W. (2020). Production of biosilica nanoparticles from biomass power plant fly ash. Waste Manag..

[71] Lee J.-W., Jang Y.-I., Park W.-S., Yun H.-D., Kim S.-W. (2020). The Effect of Fly Ash and Recycled Aggregate on the Strength and Carbon Emission Impact of FRCCs. Int. J. Concr. Struct. Mater..

[72] Bakri A.M.M.A., Kamarudin H., Bnhussain M., Nizar I.K., Mastura W.I.W. (2011). Mechanism and Chemical Reaction of Fly Ash Geopolymer Cement—A Review. Int. J. Pure Appl. Sci. Technol..

[73] Czuma N., Casanova I., Baran P., Szczurowski J., Zarębska K. (2020). CO_2_ sorption and regeneration properties of fly ash zeolites synthesized with the use of differentiated methods. Sci. Rep..

[74] Amran M., Murali G., Khalid N.H.A., Fediuk R., Ozbakkaloglu T., Lee Y.H., Haruna S., Lee Y.Y. (2021). Slag uses in making an ecofriendly and sustainable concrete: A review. Constr. Build. Mater..

[75] Bhatt A., Priyadarshini S., Mohanakrishnan A.A., Abri A., Sattler M., Techapaphawit S. (2019). Physical, chemical, and geotechnical properties of coal fly ash: A global review. Case Stud. Constr. Mater..

[76] Europe A. Global Fly Ash Market Analysis|Industry Report, 2019–2026. https://www.giiresearch.com/report/dmin776950-global-fly-ash-market.html.

[77] Sujivorakul C., Jaturapitakkul C., Taotip A. (2011). Utilization of Fly Ash, Rice Husk Ash, and Palm Oil Fuel Ash in Glass Fiber–Reinforced Concrete. J. Mater. Civ. Eng..

[78] Chindaprasirt P., Chotetanorm C., Rukzon S. (2011). Use of Palm Oil Fuel Ash to Improve Chloride and Corrosion Resistance of High-Strength and High-Workability Concrete. J. Mater. Civ. Eng..

[79] Rukzon S., Chindaprasirt P. (2009). Strength and chloride resistance of blended Portland cement mortar containing palm oil fuel ash and fly ash. Int. J. Miner. Metall. Mater..

[80] Davidovits P.J. 30 Years of Successes and Failures in Geopolymer Applications, Market Trends and Potential Breakthroughs. Proceedings of the Geopolymer 2002 Conference.

[81] Fabricius A.-L., Renner M., Voss M., Funk M., Perfoll A., Gehring F., Graf R., Fromm S., Duester L. (2020). Municipal waste incineration fly ashes: From a multi-element approach to market potential evaluation. Environ. Sci. Eur..

[82] Awal A.S.M.A., Abubakar S.I. (2011). Properties of concrete containing high volume palm oil fuel ash: Ashort-term investigation. Malays. J. Civ. Eng..

[83] Altwair N.M., Azmi M.J.M., Johari M.A.M., Fuad S.A. (2011). Strength Activity Index and Microstructural Characteristics of Treated Palm Oil Fuel Ash. Structure.

[84] Mohd Ariffin M.A., Hussin M.W., Rafique Bhutta M.A. (2011). Mix Design and Compressive Strength of Geopolymer Concrete Containing Blended Ash from Agro-Industrial Wastes. Adv. Mater. Res..

[85] Neville A.M. (1995). Properties of Concrete.

[86] Vassilev S.V., Menendez R., Alvarez D., Diaz-Somoano M., Martinez-Tarazona M.R. (2003). Phase-mineral and chemical composition of coal fly ashes as a basis for their multicomponent utilization. 1. Characterization of feed coals and fly ashes. Fuel.

[87] Barbare N., Shukla A., Bose A. (2003). Uptake and loss of water in a cenosphere-concrete composite material. Cem. Concr. Res..

[88] Rohatgi P.K., Daoud A., Schultz B.F., Puri T. (2009). Microstructure and mechanical behavior of die casting AZ91D-Fly ash cenosphere composites. Compos. Part A Appl. Sci. Manuf..

[89] Bajukov O.A., Anshits N.N., Petrov M.I., Balaev A.D., Anshits A.G. (2009). Composition of ferrospinel phase and magnetic properties of microspheres and cenospheres from fly ashes. Mater. Chem. Phys..

[90] Anshits N.N., Mikhailova O.A., Salanov A.N., Anshits A.G. (2010). Chemical composition and structure of the shell of fly ash non-perforated cenospheres produced from the combustion of the Kuznetsk coal (Russia). Fuel.

[91] Fomenko E., Anshits N., Pankova M. Fly Ash Cenospheres: Composition, Morphology, Structure, and Helium Permeability. Proceedings of the World Coal Ash Conference.

[92] Yao Z.T., Ji X.S., Sarker P.K., Tang J.H., Ge L.Q., Xia M.S., Xi Y.Q. (2015). A comprehensive review on the applications of coal fly ash. Earth-Sci. Rev..

[93] Zyrkowski M., Neto R.C., Santos L.F., Witkowski K. (2016). Characterization of fly-ash cenospheres from coal-fired power plant unit. Fuel.

[94] Siyal A.A., Azizli K.A., Ismail L., Man Z., Khan M.I. (2016). Suitability of Malaysian Fly Ash for Geopolymer Synthesis. Adv. Mater. Res..

[95] Liu F., Wang J., Qian X. (2017). Integrating phase change materials into concrete through microencapsulation using cenospheres. Cem. Concr. Compos..

[96] Wang H., Zheng K., Zhang X., Wang Y., Xiao C., Chen L., Tian X. (2018). Hollow microsphere-infused porous poly(vinylidene fluoride)/multiwall carbon nanotube composites with excellent electromagnetic shielding and low thermal transport. J. Mater. Sci..

[97] Neville A.M., Brooks J.J. (1987). Concrete Technology.

[98] Jaturapitakkul C., Tangpagasit J., Songmue S., Kiattikomol K. (2011). Filler effect and pozzolanic reaction of ground palm oil fuel ash. Constr. Build. Mater..

[99] Park S., Wu S., Liu Z., Pyo S. (2021). The Role of Supplementary Cementitious Materials (SCMs) in Ultra High Performance Concrete (UHPC): A Review. Materials.

[100] Kroehong W., Sinsiri T., Jaturapitakkul C. (2011). Effect of palm oil fuel ash fineness on packing effect and pozzolanic reaction of blended cement paste. Procedia Eng..

[101] Ibrahim M., Megat Johari M.A., Maslehuddin M., Rahman M.K., Salami B.A., Mohamed H.D. (2019). Influence of composition and concentration of alkaline activator on the properties of natural-pozzolan based green concrete. Constr. Build. Mater..

[102] Tangchirapat W., Jaturapitakkul C. (2010). Strength, drying shrinkage, and water permeability of concrete incorporating ground palm oil fuel ash. Cem. Concr. Compos..

[103] Ohenoja K., Pesonen J., Yliniemi J., Illikainen M. (2020). Utilization of fly ashes from fluidized bed combustion: A review. Sustainability.

[104] Sata V., Jaturapitakkul C., Rattanashotinunt C. (2010). Compressive Strength and Heat Evolution of Concretes Containing Palm Oil Fuel Ash. J. Mater. Civ. Eng..

[105] Awal A., Nguong S.K. A short-term investigation on high volume palm oil fuel ash (POFA) concrete. Proceedings of the 35th Conferenece on our World in Concrete and Structure.

[106] Chindaprasirt P., Rukzon S. (2009). Pore Structure Changes of Blended Cement Pastes Containing Fly Ash, Rice Husk Ash, and Palm Oil Fuel Ash Caused by Carbonation. J. Mater. Civ. Eng..

[107] Fediuk R., Mugahed Amran Y.H., Mosaberpanah M.A., Danish A., El-Zeadani M., Klyuev S.V., Vatin N. (2020). A critical review on the properties and applications of sulfur-based concrete. Materials.

[108] Malhotra V.M., Mehta P.K., Povindar K. (1996). Pozzolanic and Cementitious Materials.

[109] Chindaprasirt P., Rukzon S., Sirivivatnanon V. (2008). Resistance to chloride penetration of blended Portland cement mortar containing palm oil fuel ash, rice husk ash and fly ash. Constr. Build. Mater..

[110] Leong H.Y., Ong D.E.L., Sanjayan J.G., Nazari A. (2016). Suitability of Sarawak and Gladstone fly ash to produce geopolymers: A physical, chemical, mechanical, mineralogical and microstructural analysis. Ceram. Int..

[111] Hussin M.W., Awal A. (1996). Influence of palm oil fuel ash on strength and durability of concrete. Proceedings of the 7th International Conference on Durability of Building Materials and Components.

[112] Abdullah K., Hussin M.W., Zakaria F., Muhamad R., Abdul Hamid Z. A potential partial cement replacement material in aerated concrete. Proceedings of the 6th Asia-Pacific Structural Engineering and Construction Conference.

[113] Adam A. (2009). Strength and Durability Properties of Alkali Activated Slag and Fly Ash-Based Geopolymer Concrete. Ph.D. Thesis.

[114] Bakri A., Mustafa M., Mohammed H., Kamarudin H., Niza I.K., Zarina Y. (2011). Review on fly ash-based geopolymer concrete without Portland Cement. J. Eng. Technol. Res..

[115] Hardjito D., Wallah S.E., Sumajouw D.M.J., Rangan B.V. Introducing fly ash-based geopolymer concrete: Manufacture and engineering properties. Proceedings of the 30th Conference on Our World in Concrete and Structures.

[116] Hardjito D., Wallah S.E., Sumajouw D.M.J., Rangan B.V. (2005). Fly Ash-Based Geopolymer Concrete. Aust. J. Struct. Eng..

[117] Ryu G.S., Lee Y.B., Koh K.T., Chung Y.S. (2013). The mechanical properties of fly ash-based geopolymer concrete with alkaline activators. Constr. Build. Mater..

[118] Alsubari B., Shafigh P., Ibrahim Z., Jumaat M.Z. (2018). Heat-treated palm oil fuel ash as an effective supplementary cementitious material originating from agriculture waste. Constr. Build. Mater..

[119] Kumaravel S. (2014). Development of various curing effect of nominal strength Geopolymer concrete. J. Eng. Sci. Technol. Rev..

[120] Liu B., Shi J., Zhou F., Shen S., Ding Y., Qin J. (2020). Effects of steam curing regimes on the capillary water absorption of concrete: Prediction using multivariable regression models. Constr. Build. Mater..

[121] Patil A.A., Chore H., Dodeb P. (2014). Effect of curing condition on strength of geopolymer concrete. Adv. Concr. Constr..

[122] Manesh S.B., Madhukar R.W., Subhash V.P. (2012). Effect of duration and temperature of curing on compressive strength of geopolymer concrete. Int. J. Eng. Innov. Technol..

[123] Shafigh P., Jumaat M.Z., Mahmud H.B., Hamid N.A.A. (2012). Lightweight concrete made from crushed oil palm shell: Tensile strength and effect of initial curing on compressive strength. Constr. Build. Mater..

[124] (1983). Method for Making Test. Cylinders from Fresh Concrete.

[125] Koh H.B., Lee Y.L., Yeoh D. (2004). Medium Lightweight Concrete Containing Palm Oil Clinker (POC) and Palm Oil Fuel Ash (POFA).

[126] Kodur V.K.R., Yu B. (2016). Rational Approach for Evaluating Fire Resistance of FRP-Strengthened Concrete Beams. J. Compos. Constr..

[127] Zhu H., Wu G., Zhang L., Zhang J., Hui D. (2014). Experimental study on the fire resistance of RC beams strengthened with near-surface-mounted high-Tg BFRP bars. Compos. Part. B Eng..

[128] Nasvi M.C.M., Ranjith P.G., Sanjayan J., Bui H. (2014). Effect of temperature on permeability of geopolymer: A primary well sealant for carbon capture and storage wells. Fuel.

[129] (1953). Fire Tests on Building Materials and Structures.

[130] Karayannis V.G., Moustakas K.G., Baklavaridis A.N., Domopoulou A.E. (2018). Sustainable ash-based geopolymers. Chem. Eng. Trans..

[131] Silva G., Kim S., Aguilar R., Nakamatsu J. (2020). Natural fibers as reinforcement additives for geopolymers—A review of potential eco-friendly applications to the construction industry. Sustain. Mater. Technol..

[132] Amran Y.H.M., Farzadnia N., Ali A.A.A. (2015). Properties and applications of foamed concrete; A review. Constr. Build. Mater..

[133] Umar M.S., Jennings P., Urmee T. (2013). Strengthening the palm oil biomass Renewable Energy industry in Malaysia. Renew. Energy.

[134] Basri H., Mannan M., Zain M.F. (1999). Concrete using waste oil palm shells as aggregate. Cem. Concr. Res..

[135] Jumaat M.Z., Alengaram U.J., Mahmud H. (2009). Shear strength of oil palm shell foamed concrete beams. Mater. Des..

[136] Sofri L.A., Mohd Zahid M.Z.A., Isa N.F., Azizi Azizan M., Ahmad M.M., Ab Manaf M.B.H., Abdul Rahim M., Md Ghazaly Z., Abu Bakar J., Ahmran M.S.A. (2015). Performance of Concrete by Using Palm Oil Fuel Ash (POFA) as a Cement Replacement Material. Appl. Mech. Mater..

[137] Ibrahim R.K., Hamid R., Taha M.R. (2012). Fire resistance of high-volume fly ash mortars with nanosilica addition. Constr. Build. Mater..

[138] Shi C., Jiménez A.F., Palomo A. (2011). New cements for the 21st century: The pursuit of an alternative to Portland cement. Cem. Concr. Res..

[139] Islam A., Alengaram U.J., Zamin J., Bashar I.I. (2015). Usage of Palm Oil Industrial Wastes as Construction Materials. World Academy of Science, Engineering and Technology. J. Civ. Environ. Eng..

[140] Moghaddam F., Sirivivatnanon V., Vessalas K. (2019). The effect of fly ash fineness on heat of hydration, microstructure, flow and compressive strength of blended cement pastes. Case Stud. Constr. Mater..

[141] Kanadasan J., Razak H.A. (2015). Engineering and sustainability performance of self-compacting palm oil mill incinerated waste concrete. J. Clean. Prod..

[142] Kumar A., Kumar S. (2013). Development of paving blocks from synergistic use of red mud and fly ash using geopolymerization. Constr. Build. Mater..

[143] Zeyad A.M., Johari M.A.M., Alharbi Y.R., Abadel A.A., Amran Y.H.M., Tayeh B.A., Abutaleb A. (2021). Influence of steam curing regimes on the properties of ultrafine POFA-based high-strength green concrete. J. Build. Eng..

[144] Mosaberpanah M.A., Amran Y.H.M., Akoush A. (2020). Performance investigation of palm kernel shell ash in high strength concrete production. Comput. Concr..

[145] Islam M.M.U., Mo K.H., Alengaram U.J., Jumaat M.Z. (2016). Mechanical and fresh properties of sustainable oil palm shell lightweight concrete incorporating palm oil fuel ash. J. Clean. Prod..

[146] Noorvand H., Ali A.A.A., Demirboga R., Noorvand H., Farzadnia N. (2013). Physical and chemical characteristics of unground palm oil fuel ash cement mortars with nanosilica. Constr. Build. Mater..

[147] Nehdi M., Duquette J., El Damatty A. (2003). Performance of rice husk ash produced using a new technology as a mineral admixture in concrete. Cem. Concr. Res..

[148] Coppola L., Coffetti D., Crotti E. (2018). Plain and ultrafine fly ashes mortars for environmentally friendly construction materials. Sustainability.

[149] Lam N.T. (2020). Assessment of the compressive strength and strength activity index of cement incorporating fly ash. OP Conf. Ser. Mater. Sci. Eng..

[150] Scrivener K.L., John V.M., Gartner E.M. (2018). Eco-efficient cements: Potential economically viable solutions for a low-CO2 cement-based ma
terials industry. Cem. Concr. Res..

[151] Supit S.W.M., Shaikh F.U.A., Sarker P.K. (2014). Effect of ultrafine fly ash on mechanical properties of high volume fly ash mortar. Constr. Build. Mater..

[152] Zainudin A., KIong C.S., Ong P., Ching N.O.L., Nor N.H.M. (2017). Potential of Palm Oil Fuel Ash (POFA) Layers as Secondary Raw Material in Porcelain Stoneware Application. J. Mech. Eng..

[153] Ohenoja K., Wigren V., Österbacka J., Illikainen M. (2019). Applicability of Fly Ash from Fluidized Bed Combustion of Peat, Wood, or Wastes to Concrete. Waste Biomass Valorization.

[154] Payá J., Monzó J., Borrachero M.V., Peris-Mora E. (1996). Comparisons among magnetic and non-magnetic fly ash fractions: Strength development of cement-fly ash mortars. Waste Manag..

[155] Lesovik V., Chernysheva N., Fediuk R., Amran M., Murali G., de Azevedo A.R.G. (2021). Optimization of fresh properties and durability of the green gypsum-cement paste. Constr. Build. Mater..

[156] Hussin M., Ishida T. (1999). A study on basic properties of hardened concrete containing palm oil fuel ash as partial cement replacement material. Proceedings of the Annual Meeting in Materials and Construction, Summaries of Technical.

[157] Liu M.Y.J., Chua C.P., Alengaram U.J., Jumaat M.Z. (2014). Utilization of Palm Oil Fuel Ash as Binder in Lightweight Oil Palm Shell Geopolymer Concrete. Adv. Mater. Sci. Eng..

[158] Ranganath R.V., Bhattacharjee B., Krishnamoorthy S. (1998). Influence of size fraction of ponded ash on its pozzolanic activity. Cem. Concr. Res..

[159] Al-Qadri F.A., Saad A., Aldlaee A.A. (2009). Effect of some admixtures on heat of hydration reaction of cement pastes produced in Yemen, Saudi Arabia, and Egypt. J. Eng. Sci..

[160] Martin J.P., Collins R.A., Browning J.S., Biehl F.J. (1990). Properties and use of fly ashes for embankments. J. Energy Eng..

[161] Mollamahmutoǧlu M., Yilmaz Y. (2001). Potential use of fly ash and bentonite mixture as liner or cover at waste disposal areas. Environ. Geol..

[162] Prashanth J.P., Sivapullaiah P.V., Sridharan A. (2001). Pozzolanic fly ash as a hydraulic barrier in landfills. Eng. Geol..

[163] Kaniraj S.R., Gayathri V. (2003). Geotechnical behavior of fly ash mixed with randomly oriented fiber inclusions. Geotext. Geomembranes.

[164] Cokca E., Yilmaz Z. (2004). Use of rubber and bentonite added fly ash as a liner material. Waste Manag..

[165] Pandian N.S. (2004). Fly ash characterization with reference to geotechnical applications. J. Indian Inst. Sci..

[166] Chindaprasirt P., Jaturapitakkul C., Sinsiri T. (2005). Effect of fly ash fineness on compressive strength and pore size of blended cement paste. Cem. Concr. Compos..

[167] Kim B., Prezzi M., Salgado R. (2005). Geotechnical properties of fly and bottom ash mixtures for use in highway embankments. J. Geotech. Geoenviron. Eng..

[168] Goswami R.K., Mahanta C. (2007). Leaching characteristics of residual lateritic soils stabilised with fly ash and lime for geotechnical applications. Waste Manag..

[169] Wang S., Li V.C. (2007). Engineered cementitious composites with high-volume fly ash. ACI Mater. J..

[170] Mishra D.P., Das S.K. (2010). A study of physico-chemical and mineralogical properties of Talcher coal fly ash for stowing in underground coal mines. Mater. Charact..

[171] Velez I.C., Norris J.D., Choi Y.H., Loux S., Hinrichs K. (2011). 274 Effect of holding aspirated fluid from immature equine follicles on oocyte maturation and blastocyst production after intracytoplasmic sperm injection. Reprod. Fertil. Dev..

[172] Horpibulsuk S., Phetchuay C., Chinkulkijniwat A. (2012). Soil Stabilization by Calcium Carbide Residue and Fly Ash. J. Mater. Civ. Eng..

[173] Yoon M.S., Han S.J., Kim S.S. The mechanical properties of coal-ash generated in south Korea for using tide embankment material. Proceedings of the International Offshore and Polar Engineering Conference.

[174] Webb R.W., Stormont J.C., Stone M.C.S., Thomson B.M. (2014). Characterizing the unsaturated and saturated hydraulic properties of coal combustion by-products in landfills of northwestern new mexico. J. Am. Soc. Min. Reclam..

[175] Arulrajah A., Mohammadinia A., Phummiphan I., Horpibulsuk S., Samingthong W. (2016). Stabilization of Recycled Demolition Aggregates by Geopolymers comprising Calcium Carbide Residue, Fly Ash and Slag precursors. Constr. Build. Mater..

[176] Smith B.T., Howard I.L., Vahedifard F. (2018). Lightly cemented dredged sediments for sustainable reuse. Environ. Geotech..

[177] Saldanha R.B., Reddy K.R., Consoli N.C. (2019). Influence of sodium chloride on leaching behavior of fly ash stabilized with carbide lime. Constr. Build. Mater..

[178] Mailvaganam N.P. (1979). Factors Influencing Slump Loss in Flowing Concrete. Spec. Publ. Counc. Agric. Sci. Technol..

[179] Chaipanich A., Nochaiya T. (2010). Thermal analysis and microstructure of Portland cement-fly ash-silica fume pastes. J. Therm. Anal. Calorim..

[180] (2005). ASTM C 311-04 Standard Test Methods for Sampling and Testing Fly Ash or Natural Pozzolans for Use in Portland-Cement Concrete.

[181] American Society of Testing Materials (2010). ASTM C157 Standard Test Method for Length Change of Hardened Hydraulic-Cement Mortar and Concrete. Annu. B ASTM Stand..

[182] ASTM Committee (1900). ASTM D792-08 Standard Test Methods for Density and Specific Gravity (Relative Density) of Plastics by Displacement.

[183] Sathawane S.H., Vairagade V.S., Kene K.S. (2013). Combine effect of rice husk ash and fly ash on concrete by 30% cement replacement. Procedia Eng..

[184] (2015). ASTM Standard Test Method for Water-Extractable Sulfate in Hydrated Hydraulic Cement.

[185] Duan P., Yan C., Luo W., Zhou W. (2016). Effects of adding nano-TiO_2_ on compressive strength, drying shrinkage, carbonation and microstructure of fluidized bed fly ash based geopolymer paste. Constr. Build. Mater..

[186] Azevedo A.R.G., Vieira C.M.F., Ferreira W.M., Faria K.C.P., Pedroti L.G., Mendes B.C. (2020). Potential use of ceramic waste as precursor in the geopolymerization reaction for the production of ceramic roof tiles. J. Build. Eng..

[187] Yliniemi J., Nugteren H., Illikainen M., Tiainen M., Weststrate R., Niinimäki J. (2016). Lightweight aggregates produced by granulation of peat-wood fly ash with alkali activator. Int. J. Miner. Process..

[188] Konečný P., Ghosh P., Hrabová K., Lehner P., Teplý B. (2020). Effective methodology of sustainability assessment of concrete mixtures. Mater. Struct..

[189] De Lomas M.G., De Rojas M.I.S., Frías M., De Rojas M.I.S. (2007). Pozzolanic reaction of a spent fluid catalytic cracking catalyst in FCC-cement mortars. J. Therm. Anal. Calorim..

[190] Chandara C., Sakai E., Azizli K.A.M., Ahmad Z.A., Hashim S.F.S. (2010). The effect of unburned carbon in palm oil fuel ash on fluidity of cement pastes containing superplasticizer. Constr. Build. Mater..

[191] Memon F.A., Nuruddin M.F., Khan S., Shafiq N., Ayub T. (2013). Effect of sodium hydroxide concentration on fresh properties and compressive strength of self-compacting geopolymer concrete. J. Eng. Sci. Technol..

[192] Lesovik V., Popov D., Fediuk R., Glagolev E., Yoo D.-Y. (2020). Improvement of Mechanical and Durability Behaviors of Textile Concrete: Effect of Polymineral Composite Binders and Superabsorbent Polymers. J. Mater. Civ. Eng..

[193] Chernyshova N., Lesovik V., Fediuk R., Timokhin R. (2020). Enhancement of fresh properties and performances of the eco-friendly gypsum-cement composite (EGCC). Constr. Build. Mater..

[194] Reddy C.J., Elavenil S. (2017). Geopolymer concrete with self-compacting: A review. Int. J. Civ. Eng. Technol..

[195] Sata V., Sathonsaowaphak A., Chindaprasirt P. (2012). Resistance of lignite bottom ash geopolymer mortar to sulfate and sulfuric acid attack. Cem. Concr. Compos..

[196] Alsubari B., Shafigh P., Ibrahim Z., Alnahhal M.F., Jumaat M.Z. (2018). Properties of eco-friendly self-compacting concrete containing modified treated palm oil fuel ash. Constr. Build. Mater..

[197] Tolstoy A., Lesovik V., Fediuk R., Amran M., Gunasekaran M., Vatin N., Vasilev Y. (2020). Production of greener high-strength concrete using russian quartz sandstone mine waste aggregates. Materials.

[198] Safiuddin M., Salam M.A., Jumaat M.Z. (2013). Key Fresh Properties of Self-Consolidating High-Strength POFA Concrete. J. Mater. Civ. Eng..

[199] Volodchenko A.A., Lesovik V.S. Effective Composites Employing Fast-Hardening Gypsum Cement Binders for Additive Manufacturing. Proceedings of the International Conference “Actual Issues of Mechanical Engineering” (AIME).

[200] Awal A.S.M.A., Shehu I.A. (2013). Evaluation of heat of hydration of concrete containing high volume palm oil fuel ash. Fuel.

[201] Ranjbar N., Behnia A., Alsubari B., Birgani P.M., Jumaat M.Z. (2016). Durability and mechanical properties of self-compacting concrete incorporating palm oil fuel ash. J. Clean. Prod..

[202] Matar P., Barhoun J. (2020). Effects of waterproofing admixture on the compressive strength and permeability of recycled aggregate concrete. J. Build. Eng..

[203] Volodchenko A.A., Lesovik V.S., Cherepanova I.A., Volodchenko A.N., Zagorodnjuk L.H., Elistratkin M.Y. (2018). Peculiarities of non-autoclaved lime wall materials production using clays. Proceedings of the IOP Conference Series: Materials Science and Engineering.

[204] Awal A.S.M.A., Mohammadhosseini H. (2016). Green concrete production incorporating waste carpet fiber and palm oil fuel ash. J. Clean. Prod..

[205] Awal A.S.M.A., Shehu I.A., Ismail M. (2015). Effect of cooling regime on the residual performance of high-volume palm oil fuel ash concrete exposed to high temperatures. Constr. Build. Mater..

[206] (2005). Type III Cement May Be Used, Subject to Written Approval of The Engineer.

[207] Elistratkin M.Y., Lesovik V.S., Zagorodnjuk L.H., Pospelova E.A., Shatalova S.V. New point of view on materials development. Proceedings of the IOP Conference Series: Materials Science and Engineering.

[208] Antunes P. Performance analysis of fly ash in two-component grouts. Proceedings of the Proceedings—Rapid Excavation and Tunneling Conference.

[209] Provis J.L., Bernal S.A. (2014). Geopolymers and Related Alkali-Activated Materials. Annu. Rev. Mater. Res..

[210] Ding Y., Dai J.G., Shi C.J. (2016). Mechanical properties of alkali-activated concrete: A state-of-the-art review. Constr. Build. Mater..

[211] Tangchirapat W., Jaturapitakkul C., Chindaprasirt P. (2009). Use of palm oil fuel ash as a supplementary cementitious material for producing high-strength concrete. Constr. Build. Mater..

[212] Singh M., Siddique R. (2013). Effect of coal bottom ash as partial replacement of sand on properties of concrete. Resour. Conserv. Recycl..

[213] Prabhu R., Anuradha R., Vivek S. (2016). Experimental Research on Triple Blended Self-Compacting Geo Polymer Concrete. Asian J. Eng. Appl. Technol..

[214] Bessmertnyi V.S., Lesovik V.S., Krokhin V.P., Puchka O.V., Nikiforova E.P. (2001). The reducing effect of argon in the plasma treatment of high-melting nonmetallic materials (a review). Glass Ceram..

[215] Han Q., Wang L., Xu J. (2015). Experimental research on mechanical properties of transverse enhanced and high-temperature-resistant CFRP tendons for prestressed structure. Constr. Build. Mater..

[216] Galau D., Ismai M. (2010). Characterization of Palm Oil Fuel Ash (POFA) from Different Mill as Cement Replacement Materials.

[217] Provis J.L. (2018). Alkali-activated materials. Cem. Concr. Res..

[218] Jong L.Y., Teo D.C.L. (2014). Concrete Containing Palm Oil Fuel Ash (POFA) and Oil Palm Shell (OPS) Subjected to Elevated Temperatures. J. Civ. Eng. Sci. Technol..

[219] Chernysheva N., Lesovik V., Fediuk R., Vatin N. (2020). Improvement of Performances of the Gypsum-Cement Fiber Reinforced Composite (GCFRC). Materials.

[220] Singh N.B., Middendorf B. (2020). Geopolymers as an alternative to Portland cement: An overview. Constr. Build. Mater..

[221] Zagorodnyuk L., Lesovik V.S., Sumskoy D. (2018). Thermal insulation solutions of the reduced density. Constr. Mater. Prod..

[222] Hussin M.W., Muthusamy K., Zakaria F. (2010). Effect of Mixing Constituent toward Engineering Properties of POFA Cement-Based Aerated Concrete. J. Mater. Civ. Eng..

[223] Munir A., Abdullah, Huzaim, Sofyan, Irfandi, Safwan (2015). Utilization of Palm Oil Fuel Ash (POFA) in Producing Lightweight Foamed Concrete for Non-structural Building Material. Procedia Eng..

[224] Abid S.R., Murali G., Amran M., Vatin N., Fediuk R., Karelina M. (2021). Evaluation of mode II fracture toughness of hybrid fibrous geopolymer composites. Materials.

[225] Hussin M.W., Awal A.S.M.A. (1998). Influence of Palm Oil Fuel Ash on Sulfate Resistance of Mortar and Concrete. Spec. Publ..

[226] Fediuk R.S., Ibragimov R.A., Lesovik V.S., Pak A.A., Krylov V.V., Poleschuk M.M., Stoyushko N.Y., Gladkova N.A. (2018). Processing equipment for grinding of building powders. Proceedings of the IOP Conference Series: Materials Science and Engineering.

[227] Zhuang X.Y., Chen L., Komarneni S., Zhou C.H., Tong D.S., Yang H.M., Yu W.H., Wang H. (2016). Fly ash-based geopolymer: Clean production, properties and applications. J. Clean. Prod..

[228] Joseph B., Mathew G. (2012). Influence of aggregate content on the behavior of fly ash based geopolymer concrete. Sci. Iran..

[229] Boonserm K., Sata V., Pimraksa K., Chindaprasirt P. (2012). Improved geopolymerization of bottom ash by incorporating fly ash and using waste gypsum as additive. Cem. Concr. Compos..

[230] Li Z., Chen R., Zhang L. (2013). Utilization of chitosan biopolymer to enhance fly ash-based geopolymer. J. Mater. Sci..

[231] Saravanan G., Jeyasehar C.A., Kandasamy S. (2013). Flyash based geopolymer concrete-A state of the art review. J. Eng. Sci. Technol. Rev..

[232] Rautray S.S., Das M.R. (2018). Self-compacting geo polymer concrete: An emerging material for sustainable construction. J. Adv. Res. Dyn. Control. Syst..

[233] Ranjbar N., Mehrali M., Alengaram U.J., Metselaar H.S.C., Jumaat M.Z. (2014). Compressive strength and microstructural analysis of fly ash/palm oil fuel ash based geopolymer mortar under elevated temperatures. Constr. Build. Mater..

[234] Zhang M., El-Korchi T., Zhang G., Liang J., Tao M. (2014). Synthesis factors affecting mechanical properties, microstructure, and chemical composition of red mud-fly ash based geopolymers. Fuel.

[235] Ogundiran M.B., Nugteren H.W., Witkamp G.J. (2013). Immobilisation of lead smelting slag within spent aluminate-fly ash based geopolymers. J. Hazard. Mater..

[236] Islam A., Alengaram U.J., Jumaat M.Z., Bashar I.I. (2014). The development of compressive strength of ground granulated blast furnace slag-palm oil fuel ash-fly ash based geopolymer mortar. Mater. Des..

[237] Jun Y., Oh J.E. (2014). Mechanical and microstructural dissimilarities in alkali-activation for six Class F Korean fly ashes. Constr. Build. Mater..

[238] Awal A.S.M.A., Hussin M.W. (2011). Effect of palm oil fuel ash in controlling heat of hydration of concrete. Procedia Eng..

[239] Mohammadhosseini H., Awal A.S.M.A., Ehsan A.H. (2015). Influence of palm oil fuel ash on fresh and mechanical properties of self-compacting concrete. Sadhana.

[240] Mohammadhosseini H., Yatim J.M. (2017). Microstructure and residual properties of green concrete composites incorporating waste carpet fibers and palm oil fuel ash at elevated temperatures. J. Clean. Prod..

[241] Mostafa N.Y., Brown P.W. (2005). Heat of hydration of high reactive pozzolans in blended cements: Isothermal conduction calorimetry. Thermochim. Acta.

[242] Makul N., Fediuk R., Amran M., Zeyad A.M., Murali G., Vatin N., Klyuev S., Ozbakkaloglu T., Vasilev Y. (2021). Use of recycled concrete aggregates in production of green cement-based concrete composites: A review. Crystals.

[243] Murali G., Abid S.R., Abdelgader H.S., Amran Y.H.M., Shekarchi M., Wilde K. (2021). Repeated Projectile Impact Tests on Multi-Layered Fibrous Cementitious Composites. Int. J. Civ. Eng..

[244] Krigg P., Lamond J., Pielert J. (2006). Significance of Tests and Properties of Concrete and Concrete-Making Materials.

[245] Segui P., Aubert J.E., Husson B., Measson M. (2012). Characterization of wastepaper sludge ash for its valorization as a component of hydraulic binders. Appl. Clay Sci..

[246] Alomayri T., Shaikh F.U.A., Low I.M. (2014). Synthesis and mechanical properties of cotton fabric reinforced geopolymer composites. Compos. Part. B Eng..

[247] Zeyad A.M., Johari M.A.M., Tayeh B.A., Yusuf M.O. (2016). Efficiency of treated and untreated palm oil fuel ash as a supplementary binder on engineering and fluid transport properties of high-strength concrete. Constr. Build. Mater..

[248] Shaikh F.U.A. (2014). Effects of alkali solutions on corrosion durability of geopolymer concrete. Adv. Concr. Constr..

[249] Mohammed B.S., Al-Ganad M.A., Abdullahi M. (2011). Analytical and experimental studies on composite slabs utilising palm oil clinker concrete. Constr. Build. Mater..

[250] (2004). ASTM Standard Test. Method for Splitting Tensile Strength of Cylindrical Concrete Specimens C496/C496M-04.

[251] Dhakal S. (2009). Urban energy use and carbon emissions from cities in China and policy implications. Energy Policy.

[252] Kiattikomol K., Jaturapitakkul C., Songpiriyakij S., Chutubtim S. (2001). A study of ground coarse fly ashes with different finenesses from various sources as pozzolanic materials. Cem. Concr. Compos..

[253] Zeyad A.M., Johari M.A.M., Tayeh B.A., Yusuf M.O. (2017). Pozzolanic reactivity of ultrafine palm oil fuel ash waste on strength and durability performances of high strength concrete. J. Clean. Prod..

[254] Fediuk R., Yushin A. Composite binders for concrete with reduced permeability. Proceedings of the IOP Conference Series: Materials Science and Engineering.

[255] Haridharan M.K., Matheswaran S., Murali G., Abid S.R., Fediuk R., Amran Y.H.M., Abdelgader H.S. (2020). Impact response of two-layered grouted aggregate fibrous concrete composite under falling mass impact. Constr. Build. Mater..

[256] Alengaram U.J., Mahmud H., Jumaat M.Z. (2010). Comparison of mechanical and bond properties of oil palm kernel shell concrete with normal weight concrete. Int. J. Phys. Sci..

[257] Ayzenshtadt A., Lesovik V., Frolova M., Tutygin A., Danilov V. (2015). Nanostructured wood mineral composite. Procedia Eng..

[258] Sata V., Jaturapitakkul C., Kiattikomol K. (2004). Utilization of Palm Oil Fuel Ash in High-Strength Concrete. J. Mater. Civ. Eng..

[259] Siler P., Kratky J., De Belie N. (2012). Isothermal and solution calorimetry to assess the effect of superplasticizers and mineral admixtures on cement hydration. J. Therm. Anal. Calorim..

[260] Chandara C., Azizli K.A.M., Ahmad Z.A., Hashim S.F.S., Sakai E. (2012). Heat of hydration of blended cement containing treated ground palm oil fuel ash. Constr. Build. Mater..

[261] Karagiannis N., Karoglou M., Bakolas A., Moropoulou A. (2016). Building Materials Capillary Rise Coefficient: Concepts, Determination and Parameters Involved Authors Authors and affiliations. New Approaches Build. Pathol. Durab..

[262] Hope B.B., Page J.A., Ip A.K.C. (1986). Corrosion rates of steel in concrete. Cem. Concr. Res..

[263] Nasir V., Karimipour H., Taheri-Behrooz F., Shokrieh M.M. (2012). Corrosion behaviour and crack formation mechanism of basalt fibre in sulphuric acid. Corros. Sci..

[264] Wolsiefer J.T. (1991). Silica Fume Concrete: A Solution to Steel Reinforcement Corrosion in Concrete. Spec. Publ..

[265] Marsh B. (1984). Relationships between Engineering Properties and Microstructural Characteristics of Hardened Cement Paste Containing Pulverised-Fuel Ash as A Partial Cement Replacement. Ph.D. Thesis.

[266] Hussin M.W., Awal A.S.A.M. (1997). Palm oil fuel ash: A potential pozzolanic material in concrete construction. J. Ferrocem..

[267] Lim S.K., Tan C.S., Lim O.Y., Lee Y.L. (2013). Fresh and hardened properties of lightweight foamed concrete with palm oil fuel ash as filler. Constr. Build. Mater..

[268] Anuradha R., Thirumala R., John P.N. (2014). Optimization of molarity on workable self-compacting geopolymer concrete and strength study on SCGC by replacing fly ash with silica fume and GGBFS. Int. J. Adv. Struct. Geotech. Eng..

[269] Mannan M.A., Ganapathy C.U. (2002). Engineering properties of concrete with oil palm shell as coarse aggregate. Constr. Build. Mater..

[270] Snelson D.G., Wild S., O’Farrell M. (2008). Heat of hydration of Portland Cement-Metakaolin-Fly ash (PC-MK-PFA) blends. Cem. Concr. Res..

[271] Sideris K.K., Savva A.E., Papayianni J. (2006). Sulfate resistance and carbonation of plain and blended cements. Cem. Concr. Compos..

[272] Hong D.L.H., Mohammed B.S., Al-Fakih A., Wahab M.M.A., Liew M.S., Mugahed Amran Y.H. (2020). Deformation properties of rubberized ecc incorporating nano graphene using response surface methodology. Materials.

[273] Triantafillou T.C. (2016). Textile Fibre Composites in Civil. Engineering, 1^st^ edition.

[274] Flower D.J.M., Sanjayan J.G. (2007). Greenhouse gas emissions due to concrete manufacture. Int. J. Life Cycle Assess..

[275] Fediuk R., Pak A., Kuzmin D. (2017). Fine-Grained Concrete of Composite Binder. Proceedings of the IOP Conference Series: Materials Science and Engineering.

[276] Ren X., Qu R., Liu S., Zhao H., Wu W., Song H., Zheng C., Wu X., Gao X. (2020). Synthesis of zeolites from coal fly ash for the removal of harmful gaseous pollutants: A review. Aerosol Air Qual. Res..

